# PERIOD 2 regulates low-dose radioprotection via PER2/pGSK3β/β-catenin/Per2 loop

**DOI:** 10.1016/j.isci.2022.105546

**Published:** 2022-11-09

**Authors:** Aris T. Alexandrou, Yixin Duan, Shanxiu Xu, Clifford Tepper, Ming Fan, Jason Tang, Jonathan Berg, Wassim Basheer, Tyler Valicenti, Paul F. Wilson, Matthew A. Coleman, Andrew T. Vaughan, Loning Fu, David J. Grdina, Jefferey Murley, Aijun Wang, Gayle Woloschak, Jian Jian Li

**Affiliations:** 1 Department of Radiation Oncology, University of California at Davis, 4501 X Street, Sacramento, CA 95817, USA; 2 Department of Natural and Quantitative Sciences, Holy Cross College, Notre Dame, IN 46556, USA; 3 Department of Surgery, School of Medicine, University of California at Davis, Sacramento, CA 95817, USA; 4 Department of Biochemistry and Molecular Medicine, University of California at Davis, Sacramento, CA 95817, USA; 5 Department of Molecular and Cellular Biology, Baylor College of Medicine, Houston, TX, USA; 6 Department of Radiation and Cellular Oncology, University of Chicago, Chicago, IL 60637, USA; 7 Department of Radiation Oncology, Feinberg School of Medicine, Northwestern University, Chicago, IL 60637, USA; 8 NCI-designated Comprehensive Cancer Center, University of California at Davis, 4501 X Street, Sacramento, CA 95817, USA

**Keywords:** Biological sciences, cell biology, molecular biology

## Abstract

During evolution, humans are acclimatized to the stresses of natural radiation and circadian rhythmicity. Radiosensitivity of mammalian cells varies in the circadian period and adaptive radioprotection can be induced by pre-exposure to low-level radiation (LDR). It is unclear, however, if clock proteins participate in signaling LDR radioprotection. Herein, we demonstrate that radiosensitivity is increased in mice with the deficient Period 2 gene (Per2^def^) due to impaired DNA repair and mitochondrial function in progenitor bone marrow hematopoietic stem cells and monocytes. Per2 induction and radioprotection are also identified in LDR-treated Per2^wt^ mouse cells and in human skin (HK18) and breast (MCF-10A) epithelial cells. LDR-boosted PER2 interacts with pGSK3β(S9) which activates β-catenin and the LEF/TCF mediated gene transcription including Per2 and genes involved in DNA repair and mitochondrial functions. This study demonstrates that PER2 plays an active role in LDR adaptive radioprotection via PER2/pGSK3β/β-catenin/Per2 loop, a potential target for protecting normal cells from radiation injury.

## Highlights

• Per2-deficient mice are sensitive to stress of whole-body irradiation
• Per2 is required for low-dose radiation (LDR) mediated adaptive radioprotection
• Interaction of PER2 and pGSK3β enhances Per2 transcription
• PER2/pGSK3β/β-catenin/Per2 loop regulates LDR radioprotection

## Graphical abstract

**Figure:**
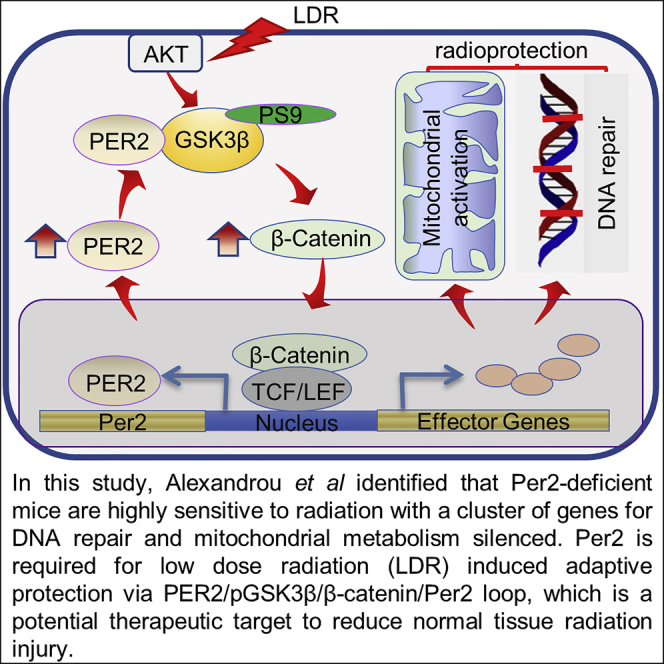

## Introduction

The biological system has developed unique adaptation ability to survive under hostile genotoxic milieu. The background natural low-dose radiation (LDR) and the circadian rhythm, both are evident on the Earth’s surface, may coordinatively contribute to the acclimating competence of mammalian cells. It has been long recognized that disturbed circadian rhythm and/or overexposure to ionizing radiation can impair cellular homeostasis leading to aging and risk of many human diseases including cancer generation.^1^^,^^2^^,^^3^^,^^4^ Animals with disturbed circadian oscillation or carrying a deficient clock protein are sensitive to radiation-induced cell injury, DNA damage response, and cell transformation potential.^5^^,^^6^^,^^7^^,^^8^^,^^9^ However, in contrast, mammalian cells exposed to naturally LDR can develop a temporary but significant cellular tolerance to subsequent genotoxic conditions such as a lethal dose of ionizing radiation.^10^^,^^11^^,^^12^^,^^13^ Elucidating the specific clock proteins in signaling the LDR-induced radiation tolerance will help to understand an integrated mechanism underlying cellular stress response.

In addition to the natural LDR which includes the radioactive sources in earth, river and atmosphere, epidemiological analysis raises a health concern on the artificial LDR such as medical diagnosis and industrial radiation applications.^14^^,^^15^^,^^16^^,^^17^^,^^18^ DNA damage response and dynamic mitochondrial metabolism, two fundamental cellular functions in cell response to genotoxic stresses are linked with circadian oscillation.^19^^,^^20^^,^^21^ Dysfunctional regulation of clock genes contributes to cell radiosensitivity and radiation-induced cancer incidence^7^ as well as the tumor chemoresistance^22^ and radioresistance.^23^ It is unclear however if such circadian sensitive radiation injuries can be compromised by LDR-induced cellular homeostasis via clock protein regulation.

The period circadian clock 2 (PER2) is a well-defined clock protein functioning in the regulation of different cellular functions and coordinating with circadian rhythmicity.^24^^,^^25^^,^^26^ PER2 is actively involved in the regulation of cell cycle progression and as a transcriptional regulator, PER2 generates a negative feedback signal to endure the circadian periodicity.^27^^,^^28^ PER2 is shown to regulate the p53 signaling pathway in DNA damage response.^19^ Studies conducted with animal models and epidemiological analyses further demonstrate that loss of PER2 function contributes to dysregulated mitochondrial metabolism in cancer initiation potential.^3^ However, how PER2 functions in signaling LDR-induced prosurvival pathways are yet to be identified.

GSK3β is a serine/threonine kinase that plays a significant role in the Wnt/β-catenin signaling pathway.^29^ GSK3β regulation is affected by circadian rhythmicity in which active GSK3β phosphorylates and activates PER2 for nuclear translocation^30^ and PER2 protein stability is regulated by active β-catenin^31^ as well as by circadian regulated cellular redox imbalance.^32^ Herein, this study provides the evidence that PER2 plays a critical role in signaling LDR-induced adaptive radioprotection. Per2 gene transcription is first enhanced by LDR and PER2 further upregulates a cluster of prosurvival genes including DNA repair and mitochondrial metabolism via PER2/pGSK3β(S9) interaction leading to active β-catenin nuclear translocation and TCF/LEF mediated gene transcription. These findings suggest that PER2/pGSK3β(S9) interaction is a potential therapeutic target in protecting normal cells from radiation injury.

## Results

### Per2^def^ mice are radiosensitive with impaired DNA repair

**Figure 1:**
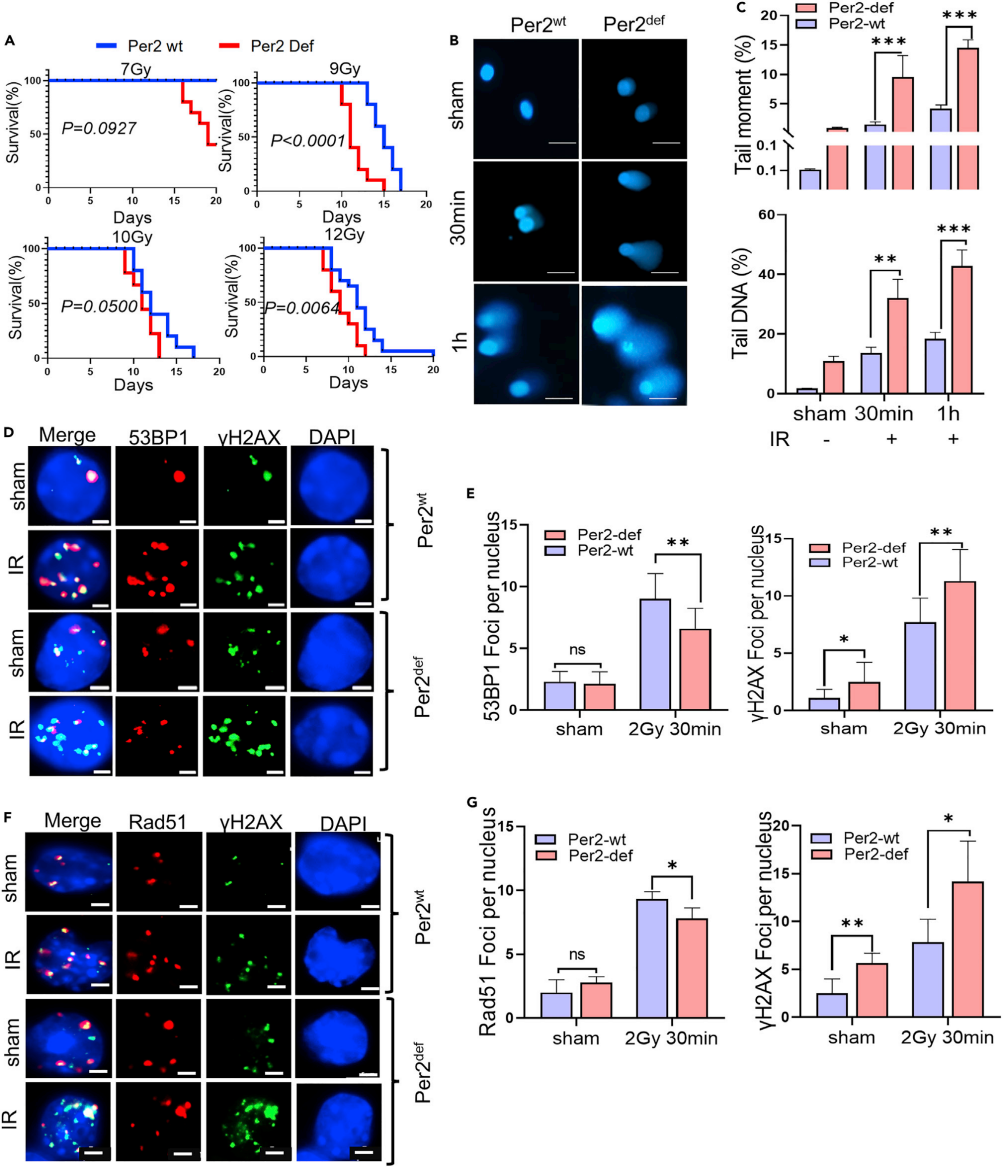
Per2^def^ mice are radiosensitive with reduced DNA repair capacity (A) Kaplan-Meier survival of Per2 wild-type (Per2^wt^, blue line) or Per2 deficient (Per2^def^, red line) C57BL/6 mice following whole body exposure to different radiation doses. WBR:7-12 Gy; Data are represented as mean ± SEM, n = 12-20/group, Kaplan-Meier survival analysis Log rank test. (B) DNA repair capacity showing representative images of alkaline comet assay of Per2^wt^ and Per2^def^ BMMNCs at indicated times (30 min or 1 h) after 1 Gy radiation; Scale bar, 50 μm. (C) Quantitation of DNA repair capacity by tailed DNA (%) and tail moment (%). Data are represented as mean ± SEM, n = 50, ∗∗p < 0.01, ∗∗∗p < 0.001, Student’s *t* test. (D) Representative images of NHEJ DNA repair capacity by 53BP1 foci analysis in Per2^wt^ and Per2^def^ BMMNCs 2 h after 2 Gy radiation; Scale bar, 2 μm. (E) Quantitation of NHEJ DNA repair capacity by counting 53BP1 foci (left) and γH2AX foci (right) per nucleus. Data are represented as mean ± SEM, n = 40, ∗p < 0.05, ∗∗p < 0.01, ns p > 0.05, Student’s *t* test. (F) Representative images of HR DNA repair capacity by Rad51 foci analysis in Per2^wt^ and Per2^def^ BMMNCs 2 h after 2 Gy radiation; Scale bar, 2 μm. (G) Quantitation of HR DNA repair capacity by counting Rad51 foci (left) and γH2AX foci (right) per nucleus. Data are represented as mean ± SEM, n = 40, ∗p < 0.05, ∗∗p < 0.01, ns p > 0.05, Student’s *t* test.

Although disturbed circadian rhythm is long recognized to raise animal radiosensitivity, it remains unclear if specific clock proteins are decisive in animal survival after radiation. In agreement with the reported radiosensitivity by disturbed circadian rhythm,^6^ it was confirmed with mice carrying a deficient form of Per2 (Per2^def^; an in-frame deletion in the PAS-B domain)^7^ compared to the counterpart C57BL/C6 Per2 wild type (Per2^wt^) mice. We found that the survival rates were significantly reduced in Per2^def^ mice following whole-body irradiation (WBI) with doses of 9, 10, and 12 Gy; whereas 7 Gy irradiation showed a less survival effect with no statistical difference was obtained (p = 0.0927, Figure 1A). These results are complementary to the report that except for accelerated aging, no difference was observed in survival and tumor incidence between Per2^def^ versus Per2^wt^ mice following WBI with 4 Gy.^33^ Thus, a threshold dose level is involved in the radiosensitivity of Per2^def^ animals. Consistent with the enhanced animal radiosensitivity, DNA damage levels were remarkedly elevated in bone marrow monocytes (BMMNCs) of Per2^def^ mice compared to Per2^wt^ mice measured by comet assay and DNA tail moment (Figures 1B and 1C). In consistence, reduced DNA damage repair ability was detected in the irradiated Per2^def^ BMMNCs compared to the Per2^wt^ BMMNCs measured by γH2AX foci with the binding of 53BP1 and Rad51 for non-homologous end-joining (NHEJ) and homologous recombination (HR) repair, respectively (Figures 1D–1G), indicating that PER2 is involved in cell radiosensitivity via DNA repair ability.

### Identification of PER2-related prosurvival genes

**Figure 2:**
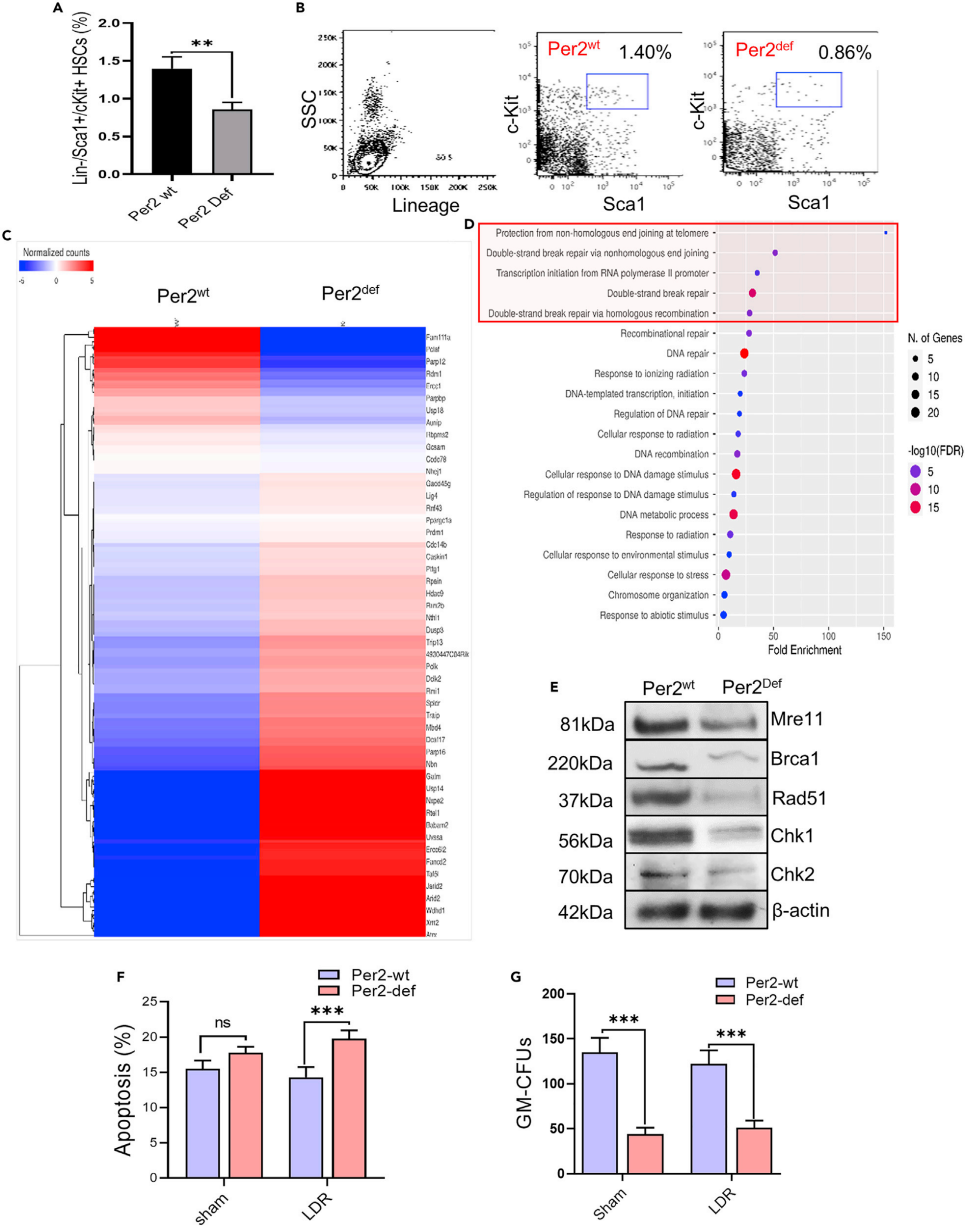
DNA repair function was impaired in Per2^def^ cells (see also Figures S1–S3) (A) Lin^−^/Sca-1^+^/c-Kit^+^ bone marrow derived progenitor hematopoietic stem cells (BM-pHSCs) were calculated by sorting of bone marrow cells in Per2^wt^ and Per2^def^ mice. Data are represented as mean ± SEM, n = 3, ∗∗p < 0.01, Student’s *t* test. (B) Representative flow cytometry sorting images of Lin^−^/Sca-1^+^/c-Kit^+^ BM-pHSCs by indicating gating region with anti-*c*-Kit and anti-Sca-l in Per2^wt^ and Per2^def^ BM-pHSCs. (C) A cluster of genes related to DNA damage repair by RNAseq analysis of Per2^wt^ versus Per2^def^ BM-pHSCs with 1.2-fold cutoff. (D) Gene ontology biological process enrichment analysis of up-regulated DNA repair genes with 1.2-fold cutoff in Per2^wt^ versus Per2^def^ Lin^−^/Sca-1^+^/c-Kit^+^ BM-pHSCs. (E) Western blot of a cluster of DNA repair factors of Mre11, Brca1, Rad51, Chk1, and Chk2 in Per2^wt^ and Per2^def^ BMMNCs. (F) The effect of LDR on apoptosis measured with flow cytometry in Per2^wt^ and Per2^def^ BMMNCs 24 h after irradiation with LDR (10 cGy). Data are represented as mean ± SEM, n = 6, ∗∗∗p < 0.001, Student’s *t* test. (G) The effect of LDR on the proliferation capacity of Per2^wt^ and Per2^def^ Lin^−^/Sca-1^+^/c-Kit^+^ BM-pHSCs was measured by GM-CFU assay. Data are represented as mean ± SEM, n = 3, ∗∗∗p < 0.01, Student’s *t* test.

RNAseq analysis was then conducted using the lineage bone marrow progenitor hematopoietic stem cells (BMpHSCs) isolated from mice Per2^wt^ and Per2^def^ bone marrow cells via sorting Lin^−^/Sca-1^+^/c-Kit^+^ population (Figure S1). Interestingly, compared to 1.40% population of Lin^−^/Sca-1^+^/c-Kit^+^ BMpHSCs detected in Per2^wt^ mice 0.86% BMpHSCs were obtained in Per2^def^ mice (Figures 2A and 2B). Using the gene ontology biological process enrichment analysis, the top 5 categories downregulated in Per2^def^ BMpHSCs were: protection from NHEJ at telomere; double-strand break (DSB) break via NHEJ; transcription initiation from RNA polymerase Ⅱ promoter, DSB repair, and DSB repair via HR (Figures 2C and 2D). Impaired DNA repair capacity was further demonstrated in Per2^def^ cells showing reduced levels of key DNA damage repair (DDR) elements, Mre11, Brca1, Rad51, Chk1, and Chk2 and other Per2 related DNA repair genes (Figures 2E and S2). In addition, the Per2^def^ BMMNCs also demonstrated a pro-apoptotic tendency in LDR and clonogenic incapability in both basal and LDR conditions (Figures 2F, 2G, and S3). These results suggest that PER2 is involved in cellular adaptive radioresistance via the regulation of prosurvival genes.

### PER2 enhances mitochondrial homeostasis

**Figure 3:**
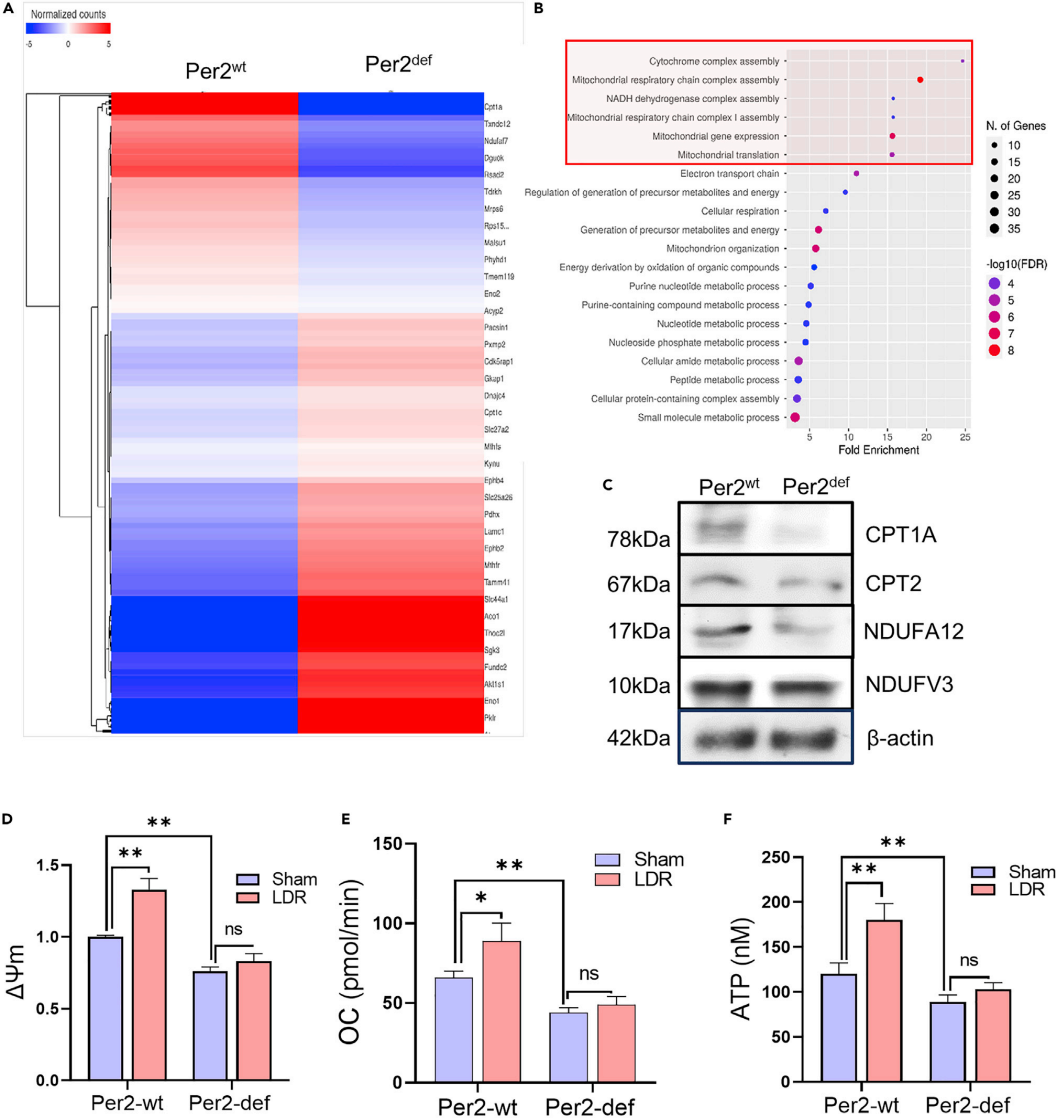
Per2 is required for LDR-induced mitochondrial activation (see also Figure S4) (A) A cluster of genes related to mitochondrial metabolic functions by RNAseq analysis of Per2^wt^ versus Per2^def^ BM-HSCs with 1.2-fold cutoff. (B) Gene ontology biological process enrichment analysis of up-regulated genes related to mitochondrial metabolic functions with 1.2-fold cutoff in Per2^wt^ versus Per2^def^ BM-pHSCs. (C–F) Western blot of a cluster of mitochondrial metabolic factors CPT1A, CPT2, NDUFA12, and NDUFV3 in Per2^wt^ and Per2^def^ BMMNCs. Mitochondrial membrane potential (D), oxygen consumption (E), and ATP generation (F) were measured in Per2^wt^ and Per2^def^ BMMNCs 24 h after LDR. Data are represented as mean ± SEM, n = 3, ∗p < 0.05; ∗∗p < 0.01, ns p > 0.05, Student’s *t* test.

Mitochondrial functions are tightly associated with nuclear genomic stability and DNA repair,^34^^,^^35^^,^^36^ which endeavored us to assume that genes involved in mitochondrial metabolism could be affected in the Per2^def^ BMHSCs (Figure 3A). The top downregulated categories in Per2^def^ BMHSCs via RNAseq profiling were: cytochrome complex assembly; mitochondrial respiratory chain complex assembly; NADH dehydrogenase complex assembly; mitochondrial respiratory chain complex I assembly and mitochondrial gene expression (Figure 3B). A group of key mitochondrial metabolism elements including CPT1A, CPT2, NDUFA12, and NDUFV3 was also identified (Figure 3C) with additional Per2-related mitochondrial metabolic genes illustrated in Figure S4. In consistence with the lack of genes in mitochondrial functions and contrasted with Per2^wt^ counterpart, Per2^def^ BMMNCs demonstrated reduced mitochondrial membrane potential (Figure 3D), oxygen consumption (OC, Figure 3E), and ATP generation (Figure 3F) without debatable LDR-induced mitochondrial adaptive metabolic activity. Together with the impaired DDR capacity shown in Figure 2, these results suggest that Per2 plays a critical role in the signaling network required for cellular adaptive response to genotoxic stress by involving DNA repair and mitochondrial metabolic functions.

### PER2 induction for low-level radiation adaptive radioprotection

**Figure 4:**
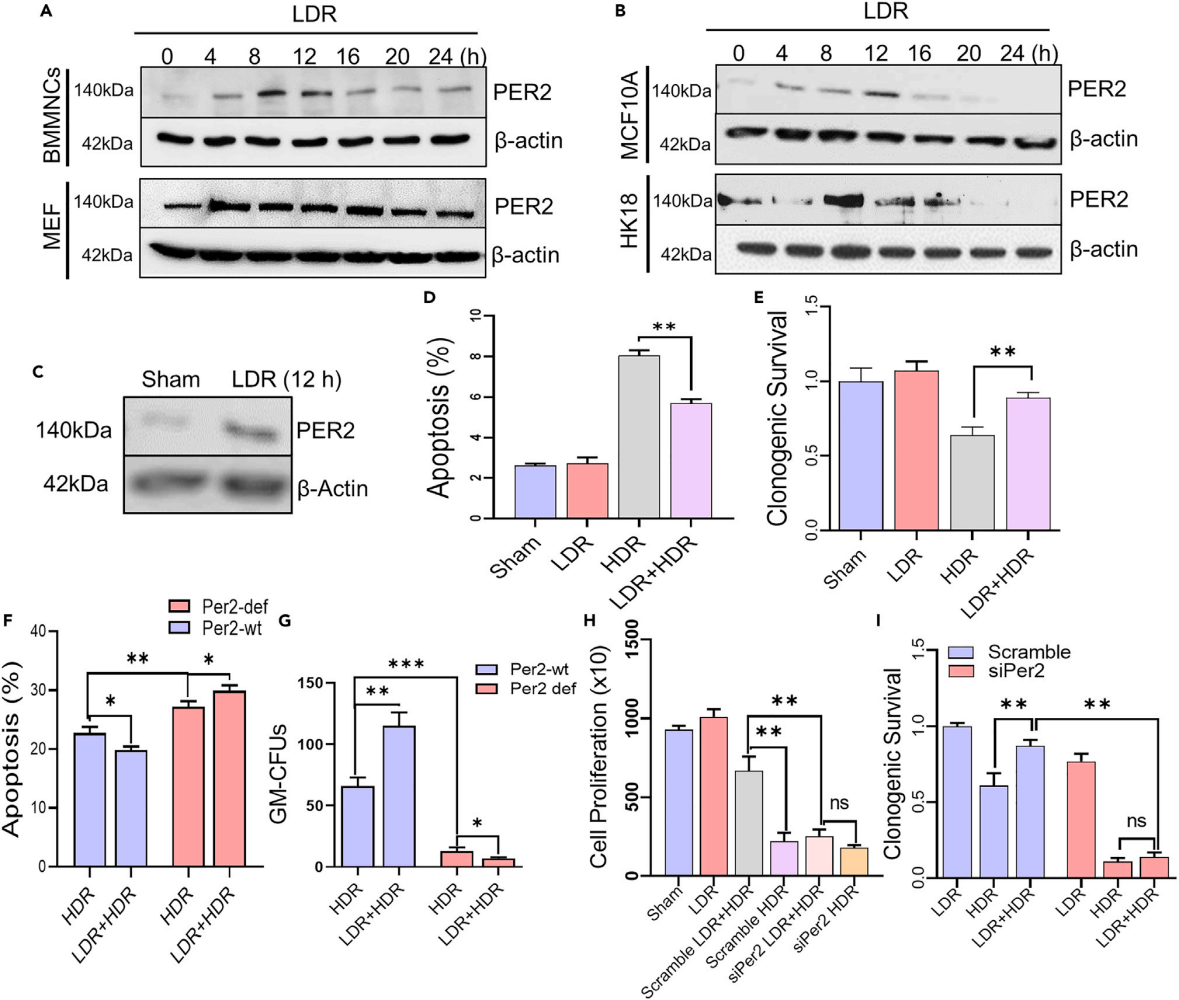
Per2 is required for LDR-induced adaptive radioprotection (see also Figure S5) (A and B). Per2 expression in mouse BMMNCs and MEFs (A) and in human mammary epithelial MCF-10A cells and skin keratinocytes HK18 (B) at different times after LDR. (C-E) Western blot of Per2 in primary cultured epithelial cells derived from healthy human mammary tissues 12 h following exposure to LDR. Cell apoptosis (D) and clonogenic survival (E) of MCF-10A cell exposed to Sham, LDR, HDR or LDR 8 h before HDR. Data are represented as mean ± SEM, n = 3; ∗∗p < 0.01, Student’s *t* test. (F) LDR-induced radioprotection was measured by apoptosis with flow cytometry in Per2^wt^ and Per2^def^ BMMNCs 24 h after HDR or LDR 8 h before HDR. Data are represented as mean ± SEM, n = 6, ∗p < 0.05, ∗∗p < 0.01, ANOVA two-way test was applied. (G–I) LDR-induced radioprotection was measured by GM-CFU assay in Per2^wt^ and Per2^def^ BMMNCs after HDR or LDR 8 h before HDR. Data are represented as mean ± SEM, n = 3, ∗p < 0.05, ∗∗p < 0.01, ∗∗∗p < 0.001, ANOVA two-way test was applied. Cell proliferation (H) and clonogenic survival (I) of MCF-10A cells transfected with siPer2 or scrambled siPer2 following exposure to Sham, LDR, HDR or LDR 8 h before HDR. Data are represented as mean ± SEM, n = 3, ∗∗p < 0.01, ns p > 0.05, ANOVA two-way test was applied.

To test if Per2 is necessary in LDR-induced adaptive radioprotection, we observed that enhanced Per2 expression was detected in Per2^wt^ BMMNCs, mouse embryonic fibroblasts (MEFs), human mammary epithelial (MCF-10A), and human skin keratinocytes (HK18) at different times following LDR (Figures 4A and 4B). Per2 was enhanced with a peak time around 8-12 h after LDR in BMMNCs, MCF-10A, and HK18 cells and a consistent raised level starting at 4 h in MEF. In addition, LDR-enhanced Per2 expression was identified in the primary cultured mammary epithelial cells isolated from a healthy woman with breast reduction surgery (Figure 4C). LDR-mediated radioprotection was recaptured in MCF-10A cells using established protocol^12^^,^^37^ of LDR before exposure to genotoxic high dose radiation (HDR, 5 Gy) measured by apoptosis and clonogenic survival (Figures 4D, 4E, and S5A). To further determine that Per2 participates in LDR-induced adaptive radioprotection, Per2^wt^ and Per2^def^ BMMNCs were exposed to LDR with or without the challenging HDR. Per2^wt^ cells showed the adaptive radioprotection with decreased apoptosis and enhanced GM-CFUs comparing to cells without LDR pre-exposure, however it was impaired in Per2^def^ BMMNCs (Figures 4F, 4G, and S5B). A specific human Per2 siRNA with 30 nM effectively blocked Per2 expression contrasted with the scrambled control (Figures S5C and S5D) and eliminated LDR-mediated cellular adaptive radioprotection in MCF-10A cells measured by cell proliferation and clonogenic survival (Figures 4H and 4I), indicating that PER2 is able to initiate a specific signaling pathway in adaptive radioprotection of cells.

### PER2 activates GSK3β/β-catenin pathway with Per2 expression

**Figure 5:**
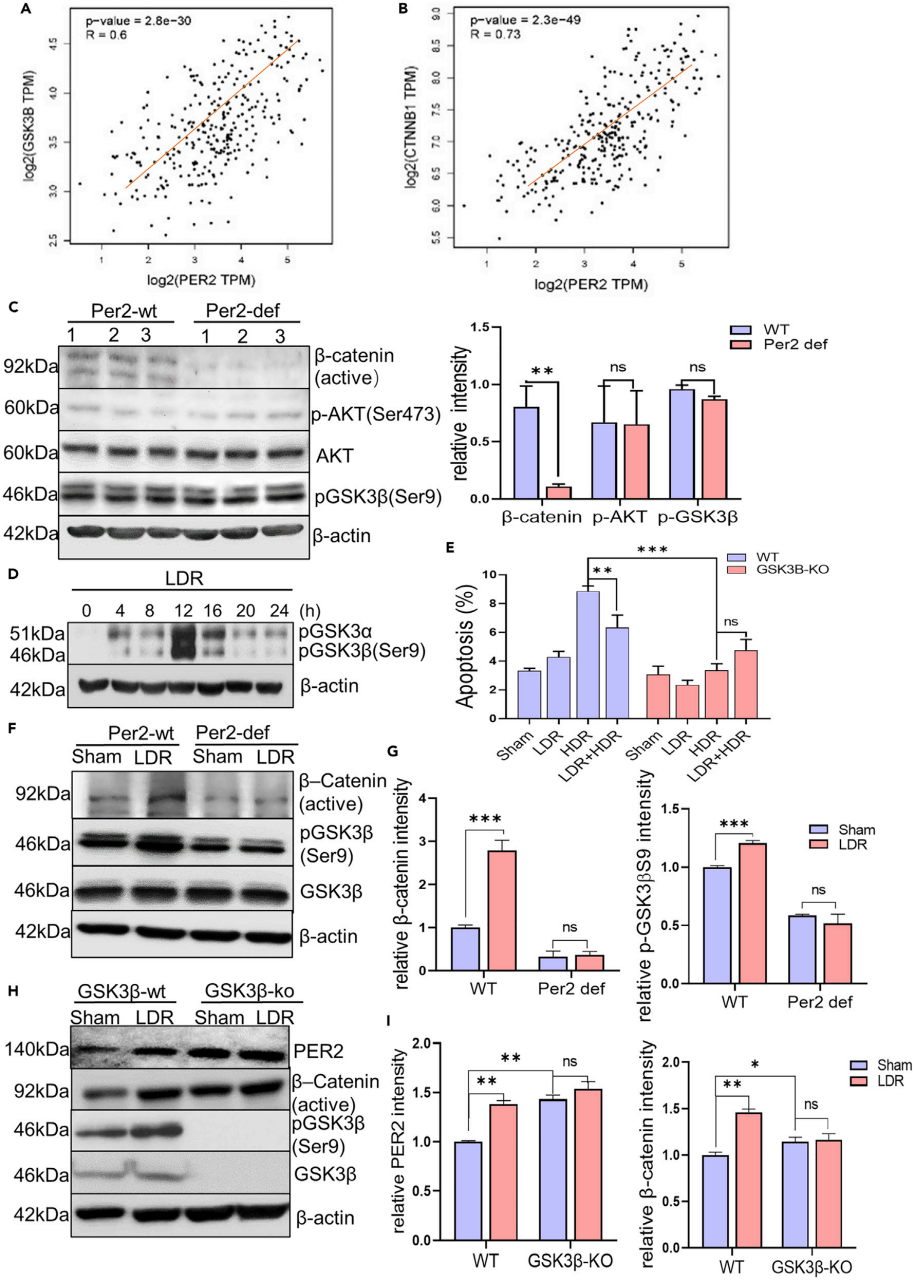
GSK3β/β-catenin pathway is involved in Per2 mediated adaptive radioprotection (see also Figure S6) (A) Correlation of Per2 and GSK3β in human mammalian tissue analyzed with the GEPIA database. Pearson correlation analysis, R = Pearson correlation coefficient. (B) Correlation of Per2 and β-catenin in human mammalian tissue analyzed with the GEPIA database. Pearson correlation analysis, R = Pearson correlation coefficient. (C) Left, western blot of phosphorylated GSK3β (Ser9), AKT, *p*-AKT and active β-catenin in Per2^wt^ and Per2^def^ BMMNCs; right, relative expression of indicated proteins quantified with ImageJ and normalized with β-actin levels. Data are represented as mean ± SEM, n = 3, ∗∗p < 0.01, ns p > 0.05, Student’s *t* test. (D) Western blot of phosphorylated GSK3β (Ser9) in Per2^wt^/GSKß^wt^ MEFs following exposure to LDR with estimated GSK3β phosphorylation peak at 12 h. (E) Cell apoptosis of Per2^wt^/GSKß^wt^ MEFs compared to Per2^wt^/GSKß^ko^ MEFs after treated with LDR (10 cGy), HDR (5 Gy), or LDR 12 h before HDR. Data are represented as mean ± SEM, n = 3, ∗∗p < 0.01, ∗∗∗p < 0.001, ns p > 0.05, ANOVA two-way test was applied. (F) Western blot of pGSK3β(Ser9), GSK3β, and active β-catenin in Per2^wt^ and Per2^def^ mice BMMNCs 12 h after sham and LDR. (G) Relative expression of pGSK3β and active β-catenin in Per2^wt^ and Per2^def^ mice BMMNCs 12 h after LDR was quantified with ImageJ and normalized with β-actin levels. Data are represented as mean ± SEM, n = 3, ∗∗∗p < 0.001, ns p > 0.05, Student’s *t* test. (H) Western blot of pGSK3β(Ser9), GSK3β, active β-catenin, and Per2 in Per2^wt^/GSK3ß^wt^ and Per2^wt^/GSK3ß^ko^ MEFs 12 h after sham and LDR. (I) Relative expression of PER2 and active β-catenin in Per2^wt^/GSK3ß^wt^ and Per2^wt^/GSK3ß^ko^ MEFs 12 h after sham and LDR was quantified with ImageJ and normalized with β-actin levels. Data are represented as mean ± SEM, n = 3, ∗p < 0.05, ∗∗p < 0.01, ns p > 0.05, ANOVA two-way test was applied.

Wnt/β-catenin pathway plays an important role in the regulation of cell proliferation and radiation response.^38^^,^^39^^,^^40^ We found that Per2 expression is positively correlated with GSK3β and β-catenin gene (CTNNB1) in the profile of human breast mammary tissue collected at GEPIA (Figures 5A and 5B). Intriguingly, although both phosphorylated AKT (*p*-AKTS473), a AKT active form and upstream regulator of GSK3β, and the phosphorylated GSK3β/ser9 (pGSK3βS9) remained unchanged in Per2^wt^ and Per2^def^ BMMNCs, active β-catenin was remarkably reduced in Per2^def^ BMMNCs (Figure 5C). These results suggest a potential critical mechanism in which PER2 controls the release of β-catenin from the GSK3β/β-catenin complex for regulating a cluster of prosurvival genes. Indeed, like Per2 induction, pGSK3β(S9) was induced with a similar peak time (8-12 h) after LDR in Per2^wt^/GSKß^wt^ MEFs following exposure to LDR (Figure 5D) with which LDR-induced adaptive radioprotection was recaptured whereas in Per2^wt^/GSK3ß^ko^ MEFs no such adaptive radioprotection was induced (Figures 5E and S6). In consistence, pGSK3β(S9) was enhanced in Per2^wt^ BMMNCs but not in Per2^def^ BMMNCs and the LDR-induced pGSK3β(S9) was accompanied by increased active form of β-catenin in Per2^wt^ BMMNCs, but not in Per2^def^ BMMNCs (Figures 5F and 5G). Since GSK3β mediated the phosphorylation of β-catenin leading to β-catenin degradation^41^ we assumed that LDR-enhanced PER2 regulates a prosurvival gene expression via β-catenin activation. As expected, in LDR-treated MCF-10A cells, the inactive form of β-catenin (pS/T β-catenin) that was enhanced at 8 h was substantially degraded at 12 h, whereas the active form of β-catenin was enhanced at 12 h (Figures S7A–S7C), which matched the peak time of *p*-GSK3β(S9) after LDR (Figure 5G). In addition, PER2, pGSK3β(S9), and active β-catenin were enhanced by LDR in GSK3β^wt^, but not detected in GSK3β^ko^ MEFs (Figures 5H and 5I). The basal level of active β-catenin and Per2 were elevated in the GSK3β^ko^ MEFs, but not enhanced by LDR, indicates that PER2-GSK3β interaction is required for LDR radioprotection (Figures 5H and 5I). Together, these results revealed that PER2/p-GSK3βS9 complex functions as a signaling switch for β-catenin activation to upregulate LDR-induced prosurvival genes.

### PER2/pGSK3β(Ser9) interaction extended pGSK3β(Ser9) life time, activating β-catenin for Per2 transcription

**Figure 6:**
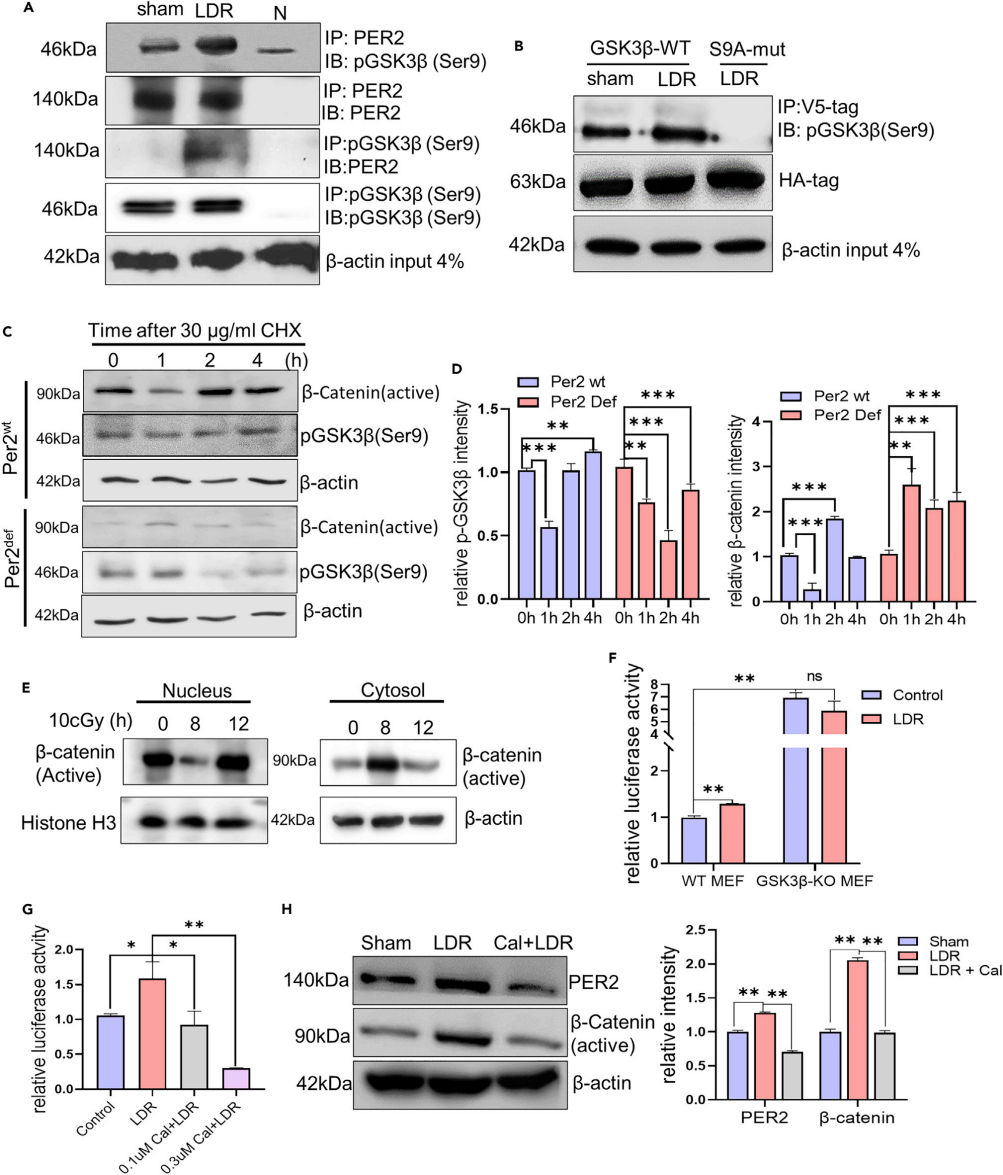
PER2 interacts with pGSK3β(Ser9) to enhance active β-catenin mediated Per2 transactivation (see also Figures S7-S9) (A) Interaction of Per2 with pGSK3β (Ser9) in Per2^wt^/GSKß^wt^ MEFs detected by immunoprecipitation 12 h after LDR followed by immunoblot with anti-pGSK3β(Ser9), or reversely, immunoprecipitation of pGSK3β(Ser9) followed by immunoblot with anti-Per2 antibody (N = negative control without antibody). (B) Immunoprecipitation of co-transfected V5-Per2 with HA-pGSK3β or HA-pGSK3β S9A-mut 293T cells 12 h following LDR. (C) Degradation of pGSK3β(Ser9) and active β-catenin measured in Per2^wt^ and Per2^def^ BMMNCs 12 h after LDR followed by cycloheximide (30 μg/ml) for indicated times. (D) Relative expression of pGSK3β(Ser9) and active β-catenin in in Per2^wt^ and Per2^def^ BMMNCs 12 h after LDR followed by cycloheximide (30 μg/ml) was quantified with ImageJ and normalized with β-actin levels. Data are represented as mean ± SEM, n = 3, ∗∗p < 0.01, ∗∗∗p < 0.001, ANOVA two-way test was applied. (E) Western blot of active β-catenin in nucleus and cytosol of Per2^wt^/GSK3ß^wt^ MEFs 8 h after LDR, using histone and β-actin as loading controls respectively for nuclear and cytosol proteins. (F) Luciferase reporter activity driven by mouse Per2 promoter in Per2^wt^/GSK3ß^wt^ MEFs compared to Per2^wt^/GSKß^ko^ MEFs 12 h after LDR; Per2 luciferase transcription activity was normalized with Renilla activity. Data are represented as mean ± SEM, n = 3, ∗∗p < 0.01, ns p > 0.05, ANOVA two-way test was applied. (G) Luciferase reporter activity driven by mouse Per2 promoter in Per2^wt^/GSK3ß^wt^ MEFs 12 h after LDR or LDR incubation with 0.1 and 0.3 μm β-catenin inhibitor Calphostin C (*Cal*, blocking β-catenin transactivation), Per2 luciferase transcription activity was normalized with Renilla activity. Data are represented as mean ± SEM, n = 3, ∗p < 0.05, ∗∗p < 0.01, ANOVA two-way test was applied. (H) Western blot of PER2 and active β-catenin in Per2^wt^/GSK3ß^wt^ MEFs 12 h after LDR or LDR with Cal (0.1 μm); right, relative expression of PER2 and active β-catenin in Per2^wt^/GSK3ß^wt^ MEFs 12 h after LDR or LDR with Cal (0.1 μm) was quantified with ImageJ and normalized with β-actin levels. Data are represented as mean ± SEM, n = 3, ∗∗p < 0.01, ANOVA two-way test was applied.

Active GSK3β is shown to phosphorylate and activate PER2 for nuclear translocation.^30^ We assumed that PER2 may directly interact with the inactive form pGSK3β(S9) and as a result activate the β-catenin/TCF-regulated prosurvival network in LDR-induced adaptive radioprotection. Indeed, although PER2/pGSK3β(S9) complex was detected in the non-LDR control, LDR strikingly enhanced the complex formation at 12 h after LDR (Figure 6A). To confirm the specificity of pGSK3β(S9) with interaction, we co-transfected the mutant pGSK3β S9A-HA and Per2-V5 plasmids into 293T cells. Co-immunoprecipitation identified PER2 interaction with wild-type GSK3β(S9), but not the S9A mutant in 293T cells with overexpressing Per2 (Figures 6B and S7D). In consistence, cycloheximide (CHX) chase assay revealed that both active form of β-catenin and pGSK3β(S9) were maintained in Per2^wt^ BMMNCs whereas active β-catenin was faintly detectable with a rapid pGSK3β(S9) degradation in Per2^def^ BMMNCs (Figures 6C and 6D), indicating that the formation of PER2/pGSK3β(S9) complex prolonged inactive form of pGSK3β(S9), enhancing active β-catenin. Furthermore, the nuclear translocated active β-catenin paralleled with reduced cytoplasmic active β-catenin in Per2^wt^/GSK3β^wt^ MEFs 12 h after LDR (Figure 6E), indicating a PER2/pGSK3β/β-catenin/TCF axis that upregulates Per2 transcription and a cluster of prosurvival genes. In Wnt/β-catenin signaling, the active β-catenin functions as a transcriptional coactivator for transcriptional factors T cell factor/lymphoid enhancer factor (TCF/LEF) with binding elements at the promoter region of the effector genes.^42^ Consistently, database searching revealed that the Per2 promoter region contains the well-defined β-catenin-TCF/LEF domains including LEF-1/TCF-1A, TCF-4, TCF-1(P), TCF-2, and TCF-3 identified in the human and mouse Per2 promoter region (Figure S8A Table A, S8B Table B). We then constructed luciferase reporters driven by the cloned Per2 promoter enriched with the TCF/LEF domains. Luciferase assay result showed enhanced reporter activity by LDR in GSK3β^wt^ MEFs, but not in the GSK3β^ko^ MEFs (Figure 6F). Moreover, LDR-induced Per2 promoter transactivation was also inhibited by Calphostin C that blocks GSK3β phosphorylation reducing the active β-catenin; Cal, 0.1 μM, 1 h; 0.3 μM, 1 h) (Figures 6G and S9A). β-catenin mediated Per2 expression was further evidenced by the increased β-catenin activity in LDR treated cells and reversed by β-catenin inhibition with calphostin C both in MEFs and MCF-10A cells (Figures 6H and S9B). Thus, LDR-induced PER2/pGSK3β/β-catenin/Per2 loop is suggested to play a major role in LDR-induced adaptive radioprotection.

### PER2 is related to β-catenin/TCF/LEF prosurvival effector genes

**Figure 7:**
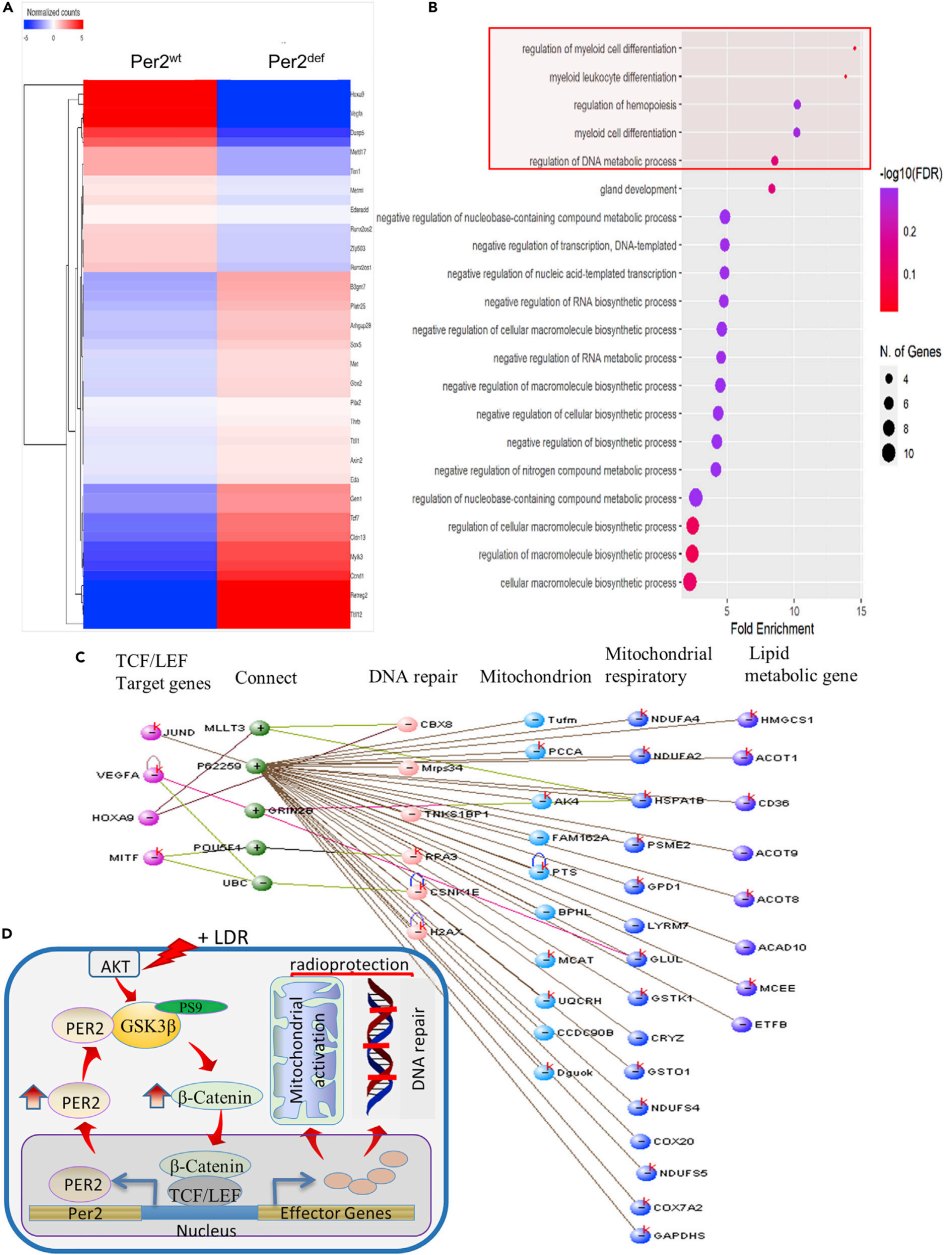
A Per2/pGSK3β/β-catenin/Per2 loop in Adaptive Radioprotection (see also Figure S10) (A) A cluster of β-catenin/TCF/LEF regulated prosurvival effector genes screened with 1.2-fold cutoff in RNAseq profile of Per2^wt^ versus Per2^def^ BMHSCs. (B) Gene ontology biological process enrichment analysis of up-regulated TCF/LEF targeted genes in Per2^wt^ versus Per2^def^ BMHSCs. (C) The interaction between four representatives upregulated TCF/LEF targeted genes (1.25-1.52-fold increased expression) with DNA repair genes and mitochondria metabolism genes in Per2^wt^ versus Per2^def^ BMpHSCs performed by VisANT, where k represents the potential KEGG pathways existing. (D) Schematic pathway of PER2/pGSK3β/β-catenin/PER2 loop in low-dose radiation-induced radioprotection. LDR-induced interaction of PER2 and pGSK3β(Ser9) leading to stabilization of pGSK3β(Ser9) enhancing active β-catenin that enters the nucleus and upregulates Per2 and a cluster of prosurvival genes involved in DNA repair and mitochondrial metabolism via β-catenin/TCF/LEF regulation. Thus, PER2/pGSK3β/β-catenin/PER2 loop may sustain the adaptive cellular homeostasis enhancing cell survival under severe genotoxic condition induced by high dose radiation.

More in-depth analysis of the Per2^wt^ versus Per2^def^ transcriptome demonstrates PER2/β-catenin boosted LDR adaptive radioresistance. Activation of Wnt/β-catenin/TCF/LEF pathway is suggested to enhance normal tissue tolerance to radiation.^43^ We attempted to define the TCF/LEF effector genes differently expressed in Per2^wt^ versus Per2^def^ BMpHSCs and revealed a cluster of PER2 related TCF effector genes (Figure 7A) indicating a short list of potential TCF regulated prosurvival genes for DDR and mitochondrial metabolism (Figure S10). The up-regulated TCF/LEF effector genes in Per2^wt^ include the regulation of DNA metabolic process, regulation of myeloid cell differentiation, hematopoiesis and macromolecule synthesis (Figure 7B). Together with other potential PER2-associated effector genes upregulated in Per2^wt^ versus Per2^def^ BMpHSCs, a prosurvival network is illustrated under upregulated LEF/TCF effector genes interaction with the cascade of upregulated DNA repair, mitochondrial functions and respiration, and lipid metabolism (Figure 7C). Altogether, our current work reveals a PER2/pGSK3β/β-catenin/Per2 cascade that is activated for cellular adaptive radioprotection induced by exposure to low-dose radiation. Under LDR, PER2 forms complex with pGSK3β to enhance β-catenin/TCF/LEF regulated effector genes including Per2 and a cluster of prosurvival target genes coordinating with DNA repair and mitochondrial metabolism for cells to survive subsequential genotoxic stresses Figure 7D).

## Discussion

Natural low-dose radiation and circadian rhythm (CR) may have coordinatively contributed to the environment-adaptive capacity in cells. This study provides experimental evidence that PER2, a fundamental factor in CR functions in signaling cellular adaptive radioprotection induced by low-dose radiation. CR is well-defined to affect cellular stress response^44^ such as CR-associated cell radiosensitivity^6^^,^^45^^,^^46^ and CR-related proliferation and differentiation of BMpHSCs.^47^ Pre-exposure of mammalian cells to LDR can generate a prosurvival advantage to subsequent genotoxic insults such as the cytotoxicity of high dose radiation.^11^^,^^12^^,^^37^^,^^48^^,^^49^ Data from circulating leukocytes of humans with simulated night shift showed circadian dysregulation of DNA repair genes and elevated DNA damage^50^ whereas cells exposed to LDR (10 cGy) initiated an adaptive cellular homeostasis raising their tolerance to subsequent more severe genotoxic stress.^12^^,^^51^^,^^52^^,^^53^^,^^54^ We report here that in addition to CR regulation, PER2 protein can function as a signaling element in LDR-induced radioprotection. A PER2/pGSK3β/β-catenin/Per2 loop is suggested by PER2 enhanced prosurvival response.

On the animal level, no difference was observed in survival and tumor incidence between Per2^def^ versus Per2^wt^ mice following WBI with a lower radiation dose (4 Gy) which was expected to accelerate aging among the survival animals.^33^ However, Fu’s^7^ research showed that Per2 deficient mice were enhanced sensitivity to radiation associated tumor development causing lowered animal survival in Per2 deficient mice. Under such experimental setting, a dose of 4 Gy radiation clearly enhances tumor formation in Per2 deficient mice indicating that Per2 deficiency is more sensitive to radiation-induced cancer risk, which is consistent with our data of whole-body irradiation with 7 Gy although the radiation doses and time period observed are different between the two experiments. The survival data in our current study further demonstrated a PER2-related animal survival advantage following WBI using a higher dose range 7-12 Gy (Figure 1A). The difference between these two animal studies may be related to the degree of DNA damages and PER2-associated repair capacity in stem cells including the DNA repair ability and mitochondrial function in the bone marrow pHSCs and monocytes.

Consistent with the Per2-related survival, Per2^def^ cell radiosensitivity is linked with an impaired DNA repair capacity with reduced BMpHSC population and mitochondrial function (Figures 1B–1G, 2A, 2B, and 3D–3F). These are supported by gene expression profiling generated from Per2^wt^ versus Per2^def^ BMpHSCs indicating a cluster of prosurvival genes involved in DNA repair and mitochondrial functions silenced in the Per2^def^ cells. Among many other potential PER2-regulated effector genes, the categories of genes involved in DNA repair and mitochondrial metabolic functions are focused based on the findings that mitochondrial dysfunction reduces the maintenance of genomic instability after radiation.^35^^,^^55^ The reduced/silenced genes in DNA damage response and mitochondrial activity appear to be the major cellular deficiencies causing the lack of LDR-induced radioprotection in Per2 deficient animals.

On the cellular level, the current study reveals that Per2^wt^ cells exhibit adaptive radioprotection induced by LDR whereas Per2^def^ cells show no radioprotection and are even sensitive to LDR-induced apoptosis with lowered clonogenic survival (Figures 2F, 2G, 4F, and 4G). In addition to DNA repair and mitochondrial homeostasis, such PER2-mediated intrinsic radioprotection may be via p53 since PER2 can directly interact with p53 to sustain a homeostatic status in genotoxic stress.^27^ Although our current study could not exclude the possibility of PER2-p53 interaction, a peak PER2 protein accumulation induced by LDR is around 8-12 h, which is probably after the peak activation of p53 reported at 4 h after LDR with 10 cGy delivered within 72 h period of time.^56^ Again, consistent with the finding from mouse cells, knockdown of Per2 in MCF-10A cells also impaired the LDR-induced survival advantages (Figures 4H and 4I). Therefore, although this work could not exclude the potential functions in cell radiosensitivity in different CR period, the clock protein PER2 itself is demonstrated to induce cellular adaptive radioprotection. Adjusting Per2 gene expression without CR adjustment may be potential effective approach to reduce cell radiosensitivity such as reducing normal tissue injury in cancer patient radiotherapy.

Wnt/β-catenin signaling is a well-defined prosurvival pathway with β-catenin functioning as the transcriptional coactivator for TCF/LEF effector gene regulation in which β-catenin is regulated following phosphorylation.^57^ Activation of Wnt/β-catenin signaling is indicated to prevent radiation damage to normal tissues leading to cell radioresistance^58^^,^^59^ and LDR-mediated Wnt/β-Catenin activation is evidenced in the proliferation of neural stem cells.^40^ In consistence with these results, our current findings further reveal a direct interaction between PER2 and pGSK3β(S9) in the Wnt/β-catenin axis. This previously unknown mechanism is shown to activate the PER2/pGSK3β/β-catenin/Per2 loop underlying PER2-promoted prosurvival advantage under genotoxic stress. Together with the defined association of PER2 with GSK3β(S9)/β-catenin via database analysis, β-catenin is reduced in the signaling pathway in Per2 deficient cells, which suggests that PER2 functions as the upstream responsory for GSK3β/β-catenin pathway for LDR-induced adaptive radioprotection.

Prominently, the PER2/pGSK3β(S9) complex may hold a fundamental biological function. An active GSK3β is shown to play a critical role in regulating pleiotropic cellular functions including the phosphorylation and nuclear transportation of PER2.^30^ We showed that following LDR, PER2 interacts with the inactive form pGSK3β(S9) to boost a temporary, but significant cellular stress adaptive status via β-catenin/TCF regulated prosurvival network. Per2^def^ BMMNCs showed a reduced β-catenin without LDR-induced β-catenin activation (Figures 5C and 5F), whereas LDR-induced pGSK3β(S9) was concurred with activation β-catenin and Per2 induction (Figures 5D and 5H). The PER2/pGSK3β/β-catenin/Per2 loop is also supported by the direct interaction of PER2 and pGSK3β(S9) and diminished by mutant pGSK3β(S9A). GSK3β-KO cells unable to induce radioprotection response by LDR (Figure 5E) and LDR enhanced reporter activity driven by mouse Per2 promoter in Per2^wt^/GSK3β^wt^ cells was dose-dependently inhibited by β-catenin inhibitor Calphostin C (Figures 6G and 6H). The PER2-Per2 feedforward activity for enhancing cell survival is also illustrated by the findings with Per2 promoter featured with multiple TCL/LEF binding sites responsible for β-catenin mediated transactivation. LEF-1 is a member of the LEF-1/T-cell-factor (TCF) family of transcription factors, a potential transcriptional regulator for β-catenin controlled Per2 transcription since LEF-1 is regulated by Wnt pathway for maintenance of cellular genomic integrity.^60^ Again, LDR-mediated Per2 promotor activation was more enhanced in WT versus GSK3β-KO cells (Figure 6F) whereas inhibiting β-catenin also suppressed Per2 promotor activity and Per2 expression. Together, these results indicate that PER2/pGSK3β(S9) complex functions to regulate LDR-induced radioprotection by β-catenin/TCF/LEF-regulated prosurvival genes (Figure 7D).

In summary, this study provides evidence indicating that the clock protein PER2 participates in radiation-induced adaptive response via PER2/pGSK3β/β-catenin/Per2 loop that upregulate a cluster of prosurvival genes including DNA repair and mitochondrial metabolism. The PER2/pGSK3β/β-catenin/Per2 cascade may be targeted to raise normal cell tolerance to radiation-induced injury.

### Limitations of the study

One limitation of this study is the uncertainty of PER2 in the radioprotection of normal human tissue. Although several human cell lines and primary human epithelial cells are indeed induced Per2 expression with radioprotection and Per2^Def^ mice showed an enhanced radiosensitivity, the PER2/pGSK3β/β-catenin/Per2 loop is not identified *in vivo* models. Thus, the conclusion of this work could be further validated by *in vivo* studies and/or pre-clinical trials with patients after receiving an equivalent diagnostic low-level irradiation. Additional studies may further elucidate if PER2/pGSK3β/β-catenin/Per2 loop is involved in a dynamic pattern in irradiated human cells regarding its function in sustaining cellular homeostasis via keeping an elevated level of prosurvival genes.

## STAR★Methods

### Key resources table

**Table:** 

REAGENT or RESOURCE	SOURCE	IDENTIFIER
**Antibodies**		

Western Blotting: Anti Mre11	Cell Signaling	Cat# 4895; RRID: AB_2145100
Western Blotting: Anti BRCA1	Cell Signaling	Cat# 9025S; RRID: AB_2734746
Western Blotting: Anti Rad51	Santa Cruz	Cat# sc-7410; RRID: AB_2177093
Western Blotting: Anti Chk1	Santa Cruz	Cat# sc-7898; RRID: AB_2229488
Western Blotting: Anti Per2	Santa Cruz	Cat# sc-25363; RRID: AB_2161685
Western Blotting: Anti total-ß-catenin	Cell Signaling	Cat# 8480; RRID: AB_11127855
Western Blotting: Anti active-ß-catenin	Cell Signaling	Cat# 8814; RRID: AB_2798251
Western Blotting: Anti β-Actin	Sigma-Aldrich	Cat# A5441; RRID: AB_476744
Western Blotting: Anti p-GSK3ß (Ser9)	Cell Signaling	Cat# 5558T; RRID: AB_2798445
Western Blotting: Anti GSK3β	Cell Signaling	Cat#9315S; RRID: AB_490890
Immunoprecipitation: Anti Per2	BD	Cat# 611138; AB_398449
Immunoprecipitation: Anti p-GSK3ß	Santa Cruz	Cat# 5558T; RRID: AB_2798445
Immunoprecipitation:l anti-V5	Proteintech	Cat# 66007-1; RRID: AB_2734694

**Chemicals, peptides, and recombinant proteins**		

RIPA Buffer (10X)	Cell Signaling	Cat# 9806S;
ECL Western blotting detection kit	Biosciences	Cat# R1100;
Fetal Bovine Serum	Corning CellGro	Cat# 35010CV
Penicillin/Streptomycin	Corning CellGro	Cat# 30-002-CI
Eagle’s MEM	Corning CellGro	Cat# 10-010-CV
High-Glucose DMEM	Corning CellGro	Cat# 15-017-CV
L-glutamine	Sigma-Aldrich	Cat# G7513
Cholera Toxin	VWR	Cat# 80055-160
Hydrocortisone	VWR	Cat# AAA16292-03
Insulin	Sigma-Aldrich	Cat# I9278
TRIzol Reagent	Invitrogen	Cat# 15596-018
Lithium Chloride	VWR	Cat# A10531-22
Melatonin	Sigma-Aldrich	Cat# M5250-1G
Dimethyl Sulfoxide(DMSO)	Sigma-Aldrich	Cat# D2650
Annexin-binding Buffer	BioSource, Invitrogen	Cat# V13246
Propidium Iodide	Sigma-Aldrich	Cat# P4864
Triton X-100	Sigma-Aldrich	Cat# X100-500ML
Bovine Serum Albumin (BSA)	Sigma-Aldrich	Cat# 9048-46-8
HEPES	Alfa Aesar	Cat# A1477718
Granulocyte-Macrophage Colony Stimulating Factor (GM-CSF)	R&D Systems	Cat# 415-ML
Hank’s Balanced Salt Solution	Gibco	Cat# 14175-095
MTT Reagent	Sigma-Aldrich	Cat# M-2128
Digitonin	Biosynth	Cat# D3200
Succinate	Alfa Aesar	Cat# AA33386-30
Luciferase Assay Reagent	Promega	Cat# E1483
EDTA	Corning CellGro	Cat# 46034CI
DTT	BIO RAD	Cat# 161-0611
Phenylmethylsulfonyl Fluoride (PMSF)	Sigma-Aldrich	Cat# P7626
Lipofectamine RNAiMAX Reagent	Invitrogen	Cat# 13778-100
propidium iodide	Invitrogen	Cat# P1304MP
Trichloroacetic Acid (TCA)	VWR	Cat# VW3928-2
Turbo Reagent Transfection	Thermo Scientific	Cat# R0531

**Critical commercial assays**		

KAPA Library Quantification Kit	Kapa Biosystems, Inc.	Cat# kk4824
Luciferase-based ATP Assay Kit	AAT Bioquest	Cat# 21617
BCA Protein Assay Kit	Pierce	Cat# 23228
Silencer siRNA Construction Kit	Ambion	Cat# AM1620
TruSeq SBS Kit v3-HS	illumina	Cat # FC-401-3001
PLX304V5	Addgene	Cat# 25890

**Software and algorithms**		

HiSeq Control Software with Real Time Analysis (HCS 1.5/RTA 1.13)		https://www.veritone.com/applications/attribute/?creative=479240006156&keyword=%2Breal-time%20%2Banalysis&matchtype=b&network=g&device=c&utm_medium=cpc&utm_campaign=Attribute&utm_source=google&utm_content=479240006156&utm_term=%2Breal-time%20%2Banalysis&gclid=EAIaIQobChMIq_7k0_fe8gIVvyCtBh3v3QIHEAAYASAAEgL28vD_BwE
CASAVA 1.8 software	Illumina	
ImageJ		https://imagej.nih.gov/ij/
TopHat software		https://www.rfpio.com/rfp-software/?utm_source=adwords&utm_medium=cpc&utm_campaign=Search-Demo&gclid=EAIaIQobChMI252hsPfe8gIVyB6tBh2PHg3PEAAYASAAEgKBcvD_BwE
Cufflinks software		http://cole-trapnell-lab.github.io/cufflinks/install/
Flowjo	Tree Star, Inc.	https://pubmed.ncbi.nlm.nih.gov/31249491
GraphPadPrism	GraphPad Software, Inc.	https://www.graphpad.com/scientific-software/prism/
CASPlab		https://casplab.com/

**Predictome database**		

Database for Annotation, Visualization and Integrated Discovery (DAVID) Bioinformatics Resources 6.7		https://david.ncifcrf.gov/summary.jsp
Illumina HiSeq 2000 sequencing system	QB3 Vincent J. Coates Genomics Sequencing Laboratory, UC Berkeley	https://www.illumina.com/documents/products/datasheets/datasheet_hiseq2000.pdf
GEPIA database		http://gepia.cancer-pku.cn/

**Recombinant DNA**		

Human Per2 cDNA vector pLenti6.3/V5-DEST	The University of Texas MD Anderson Cancer Center	https://www.addgene.org/vector-database/6089/

**Experimental models: Organisms/strains**		

C57BL/6 Per2-def	Baylor College of Medicine	Baylor College of Medicine
Per2 associated DNA repair genes	NCBI Gene Expression Omnibus (GEO)	GSE128418
RNA-Seq data of Per2Def versus Per2wt mouse cells	NCBI Gene Expression Omnibus (GEO); Li Lab	GSE128418 This paper
Per2-associated mitochondrial function genes	NCBI Gene Expression Omnibus (GEO)	GSE128418
Per2-Wt cell lines	Li Lab	This paper
Per2-Def cell lines	Li Lab	This paper
Per2 siRNA sequence set	Li Lab	This paper

**Other**		

Zeiss LSM710 confocal microscope system	ZEISS	https://www.zeiss.com/corporate/int/home.html
Turner TD20/20 Luminometer	Promega	https://www.promega.com/products/microplate-readers-fluorometers-luminometers/microplate-readers/glomax-discover-system/?catNum=GM3000&gclid=EAIaIQobChMI7afn0vbe8gIV-BitBh3CQQchEAAYAiAAEgJTAvD_BwE

### Resource Availability

#### Lead contact

Further information and requests for resources and reagents should be directed to and will be fulfilled by the Lead Contact, Jian Jian Li (jijli@ucdavis.edu).

#### Materials availability

Any additional information required to reanalyse the data reported in this paper is available from the lead contact upon request.

### Experimental model and subject details

#### Ethics statement

All animal experiments were approved by the Institutional Animal Care and Use Committee (IACUC) of University of California at Davis and operated following the NSFC regulations concerning the care and use of experimental animals. The 129/C57BL/6 of Per2^def^ founders were backcrossed to C57/B6 mice for 8–10 generations to generate C57BL/6 inbred mice and the Per2^def^ status was confirmed following the established methods.^61^^,^^62^ Three paired male and female Per2^def^ (C57BL/6) mice were transferred from Baylor College of Medicine to the University of California at Davis Medical Center animal facility and bred for *in vivo* experiments and age matched Per2^wt^ C57BL/6 mice were purchased from Charles River Laboratories. Both Per2^def^ and Per2^wt^ mice were housed 4 mice per cage at an ambient temperature of 22 ± 2°C and provided regular chow and filtered tap water *ad libitum*. Before each experiment, both mice were maintained under a 12:12 light/dark cycle for at least 2 weeks; 8–10 weeks old female mice with similar body weight (20-25g) were selected for the radiation experiment.

#### Mouse irradiation

Mouse whole body irradiation (WBI) was performed following the established protocol^63^ and the guidelines of the University of California, Davis IACUC approved protocol (#20579) using a linear accelerator at the Department of Radiation Oncology, UC Davis Cancer Center (Shimazu Seisakush Ltd., Kyoto, Japan) at 0.57 Gy/min (irradiation parameters, 200 kVp, 20 mA, with 0.5 mm Cu and 0.5 mm Al: half-value layer, 1.234-mmCu; target-skin distance 52 cm; 1Gy = 100 rad). Both Per2^def^ and Per2^wt^ mice were grouped (n = 8–20 per group), and radiation with a single dose was delivered under sleep conditions by anesthesia at the time of 6:00 p.m. After radiation mice were maintained at the regular husbandry care and monitored for radiation responses including body weight and survival rate at different time intervals following irradiation.

#### Cell line and culture

Human mammary epithelial MCF-10A cells were maintained and cultured in DMEM (Corning, Cat. 15-017-CV) with 10% horse serum, 20 ng/mLl epidermal growth factor, 100 ng/ml cholera toxin (VWR, Cat. 80055-160), 0.5 mg/ml hydrocortisone (VWR, Cat. AAA16292-03), 10 mg/ml insulin (Sigma, Cat. I9278), 1% penicillin/streptomycin (Corning, Cat. 30-002-CI) in a humidified incubator (5% CO_2_). Human skin keratinocytes (HK18), mouse embryonic fibroblasts (MEF), and HEK293T cells were maintained in DMEM supplemented with 10% fetal bovine serum and 1% penicillin/streptomycin in a humidified incubator (5% CO_2_). Cells were subcultured by trypsinization, and all experiments were done within 20–60 passages. MEF cells from wild-type and GSK3ß^−/−^ mouse embryos were retrieved from Dr. James Woodgett’s lab and were frozen at early (2–5) passages to be used for less than 4 weeks in continuous culture. All cell lines were handled in the cell culture facility according to lab safety protocols.

### Method details

#### Comet assay of BMMNCs

Comet assays were performed with BMMNCs isolated from Per2^wt^ and Per2^def^ mouse bone marrow using the Trevigen’s Comet Assay kit (Alkaline version, Trevigen Laboratory), which is more sensitive to detect smaller amounts of DNA damage including single and double-stranded breaks. Per2^wt^ and Per2^def^ BMMNCs were irradiated with 1 Gy radiation. Cells were then washed, resuspended in PBS and 10 μL of cell suspension (about 250 cells) were mixed with 90 μL 0.8% Low Melting Agarose. The mixture was added onto the circle of pre-warmed Comet Slide (R&D, Cat. 4250-050-03) and the slide was placed at 4°C for 30 min to allow agarose to form gel; then, immersed in prechilled lysis solution (10 mM Tris-HCl, pH 10, 2.5 M NaCl, 100 mM EDTA, 5% DMSO, 1% Triton X-100) for 30 min at 4°C. Excess buffer was drained from slides and immersed in freshly prepared alkaline unwinding/electrophoresis solution (300 mM NaOH, 1 mM EDTA, pH > 13) for 30 min at room temperature in the dark. The slides underwent electrophoresis at 21 V for 30 min in the cold. The separated nuclear DNA was stained with DAPI (Sigma, Cat. 10236276001), and the images of the comets were captured under a fluorescence microscope. For each sample, a minimum of 50 cells was analyzed using the software CASPlab (https://casplab.com/), and the DNA damage was represented as percent tail DNA and tail moment. The experiments were repeated twice, and control (untreated cells) was used to determine the characteristics of DNA fragmentations.

#### DNA damage foci analysis of BMMNCs

Bone marrow monocytes (BMMNCs) were purified following the established protocol by flushing out the total bone marrow cells under aseptic conditions using 27 G needles from the long bones (tibias and femurs) of mice and cultured with DMEM/F12 supplemented with 10% fetal bovine serum (FBS), 10 Mm L-Glutamine, 100 U/ml M-CSF (Peprotech, Cat. 315-02), and 1% penicillin/streptomycin for 7–10 days. BMMNCs isolated from Per2^wt^ and Per2^def^ mouse bone marrow were attached on coverslips for 30 min before irradiation with 2 Gy X-ray using a Cabinet X-ray System Faxitron Series (dose rate: 0.028 Gy/min; Hewlett Packard, McMinnville, OR, USA); then fixed in 4% paraformaldehyde for 10 min at room temperature, permeabilized in 0.2% Triton X-100 (Sigma, Cat. X100-500ML) for 10 min, and blocked in 1% BSA (BSA, Sigma, Cat. 9048-46-8) for 1 h at room temperature. For non-homologous DNA end joining (NHEJ) analysis, cells were incubated with the primary antibodies 53BP1 (Cell signaling, Cat. 4937) and γH2AX (Cell signaling, Cat. 9718) overnight at 4°C, and for homologous recombination (HR) analysis, primary antibodies Rad51 (Santa Cruz Biotechnology, sc-7410) and γH2AX were used. Cell were then incubated at room temperature with the fluorescence-conjugated secondary antibody for 1 h in the dark. Cell nuclei were then counterstained with DAPI contained in the mounting solution and the DNA repair foci images were acquired using a Zeiss LSM710 confocal microscope system and analyzed with ImageJ software. The experiment was repeated three times and at least 30–40 cells were scored for each sample. Data represent the percentage of cells containing more than three foci per nucleus.

#### RNAseq analysis of Lin^−^/Sca1^+^/cKit^+^ pHSCs

Progenitor hematopoietic stem cells (pHSCs) were sorted from mice bone marrow by FACSVantage (Becton Dickinson) for c-Kit (CD117), Sca-1, and low to negative levels of lineage markers (Lin) -CD2, -CD3ε, -CD4, -CD8α, -Ter119, -B220, -CD19, -Mac1, and -Gr1. Data parameters were collected in the list mode data file and were analyzed by the software program Flowjo. The sorted Per2^wt^ and Per2^def^ pHSCs were then pelleted by centrifugation and stored at −80°C. Total cellular RNA was isolated using the TRIzol reagent (Invitrogen, Cat. 15596-018) and also a modified protocol that incorporates an additional extraction with phenol/chloroform/isoamyl alcohol (25:24:1, pH 4.3) was included. RNA quantity and quality were assessed on a NanoDrop spectrophotometer (Thermo Scientific) and the Agilent 2100 Bioanalyzer (Agilent Technologies), respectively.

RNAseq libraries were prepared from NEBNext mRNA Library Prep Master Mix Kit and NEBNext Multiplex Oligos (New England BioLabs, Ipswich, MA) according to the manufacturer’s standard protocol. Briefly, poly-adenylated mRNA was purified from total RNA by two rounds of binding to oligo d(T)_25_ paramagnetic beads, which was then followed by mRNA fragmentation by incubation in the presence of divalent cations at 94°C for 5 min. Double-stranded cDNA was then generated by random-primed first-strand synthesis with ProtoScript II reverse transcriptase and subsequent second strand synthesis with NEBNext Second Strand Synthesis Enzyme Mix. The cDNA was then blunt-ended and 3′-dA tailed by incubation with Klenow Fragment (3′→ 5′exo-) and dATP. Illumina paired-end (PE) adapters were then ligated, followed by size selection and purification of the cDNA library with Agencourt AMPure XP beads. Libraries were then enriched and indexed by high-fidelity PCR amplification (12 cycles) with Q5 High-Fidelity DNA Polymerase (NEB) and adapter-specific and multiplex primers. Bead-purified libraries were then assessed with an Agilent 2100 Bioanalyzer and quantified with a Qubit fluorometer (Invitrogen) and qPCR using a KAPA Library Quantification Kit (Kapa Biosystems, Inc., Wilmington, MA). Indexed libraries were pooled and multiplex sequenced (1 × 100 bp, single-read, 4 libraries/lane) with an Illumina HiSeq 2000 sequencing system (QB3 Vincent J. Coates Genomics Sequencing Laboratory, UC Berkeley) using standard Illumina kitted reagents (TruSeq SBS Kit v3-HS, illumine, Cat. FC-401-3001).

#### Next generation sequencing (NGS) data analysis

Image processing, base calling, quality scoring (Phred), and sample demultiplexing were executed by HiSeq Control Software with Real Time Analysis (HCS 1.5/RTA 1.13) and CASAVA 1.8 software (Illumina; San Diego, CA). RNAseq sequence reads (FASTQ format) were aligned to the reference human genome assembly (Feb. 2009, GRCh37/hg19) using TopHat software which performs splice junction mapping after read alignment with Bowtie2. Gene- and transcript-level expression were comprehensively quantified with Cufflinks software which performed 1) transcript assembly, 2) identification of splice variants, and 3) quantification of normalized expression as FPKM (fragments per kilobase of transcript per million mapped reads) values. Differentially expressed genes in Per2^wt^ verses Per2^def^ BMMNCs were determined using the Cufflinks program. Biological interpretation of the resulting gene list was performed using functional annotation and clustering tools available at the Database for Annotation, Visualization and Integrated Discovery (DAVID) Bioinformatics Resources 6.7 with calculated enrichment scores based on a Fisher Exact Test.^64^ Subsequently, network analysis of the differentially-regulated genes was performed using VisANT tool based on functional relations in the Predictome database.^65^ Gene ontology biological process enrichment analysis was performed using the ggplot2 R language packages.

#### Western blotting

Cells were collected from the 60-mm culture dishes, washed with PBS, and lysed on ice in 100 μL of 1x RIPA buffer (Cell Signaling, Cat. 9806S) with 1x proteinase inhibitor cocktail (Roche, 11836170001) per dish. Protein concentrations were determined using a BCA Protein Assay kit (Pierce, Cat. 23228). Equal aliquots of protein (20 μg/lane) were run in SDS-PAGE and transferred by semi-dry transfer onto PVDF membranes. Then, membranes were blocked with 5% milk for 1 h and incubated with primary antibodies at 4°C overnight. After secondary antibody conjugation, the membranes were visualized by KwikQuant Imager using the ECL Western blotting detection kit (Biosciences, Cat. R1100). The primary antibody preparations against Mre11 (Cat.4895), Brca1 (Cat.9025S), total β-catenin (Cat. 8480), active β-catenin (Cat. 8814), pGSK3β (Ser9) (Cat.5558T), histone H3 (Cat. 4499s), and total GSK3β (Cat. 9315S) were purchased from Cell Signaling Technology (Danvers, MA, USA). Rad51 (Cat. sc-7410), Chk1 (Cat. sc-7898), and Per2 (Cat. sc-25363) were purchased from Santa Cruz Biotechnology Inc. (Santa Cruz, CA, USA). Chk2 (Cat. ab3292-500), CPT1A (Cat. ab128568), and NDUF12 (Cat. ab91521) were purchased from Abcam. β-actin is from Sigma (Cat. A5441).

#### Measurement of LDR induced adaptive radioprotection

Designated cells were treated with LDR (10 cGy) or HDR (5 Gy) using the Cabinet X-ray and allowed to grow for 0–24 h harvesting at 4 h intervals after LDR. For radioprotection experiments, mouse BMMNCs, MEF cells, and human MCF10A cells were irradiated with LDR (10 cGy) followed by culturing for 12 h before exposure to a single dose of HDR (5 Gy). Cells were collected 24 h after HDR for measuring apoptosis and clonogenic survival.

#### Flow cytometry analysis of apoptosis

Sham or irradiation-treated GSK3ß^wt^ MEFs, GSK3ß^ko^ MEFs, and MCF-10A were analyzed 8 h post-LDR treatment and 24 h after HDR treatment. Cells were resuspended in 500 μL of Annexin-binding buffer (Biosource, Cat. V13246) at a population of 2 x 10^6^ cells/ml. One microliter of propidium iodide was added to 100 μL of cell suspensions and incubated at 37°C for 15 min. Cell samples were analyzed on FACScan (Becton Dickinson).

#### Granulocyte/macrophage-colony formation (GM-CFU) assay

The GM-CFU assay is conducted using mouse methylcellulose complete media (R&D, cat. HSC007). The mouse bone marrow cells isolated from femurs and tibias were incubated with 1X lysis buffer (Biolegend. Cat. 420301) to lyse red blood cells (RBC). The leukocytes were then transferred into a sterile Petri dish containing IMDM (Gibco Cat. 12440-053) with 2% FBS. Cells received different radiation treatments according the experimental design (Sham, 10 cGy, 5 Gy, and 10 cGy +5 Gy). 5 × 10^4^ cells were added into 3 mL of mouse methylcellulose completed medium and the cells:medium ratio was kept in 1:10 v/v. 1.1 mL of mixture and was dispensed into 35 mm dishes that were pre-screened to ensure low cell adherence. Each dish was gently tilted and rotated to distribute methylcellulose evenly. Cells were incubated for 10 to 15 days, the colonies were counted and evaluated by using Nikon microscope (Eclipse, E1000M, Japan) and scored dishes were gridded.

#### Measuring mitochondria membrane potential

Irradiated bone marrow cells were incubated with 2 mg/mL of JC-1 for 30 min. A plate reader (Spectra Max M2e, Molecular Devices Co.) measured the fluorescence intensity of the red precipitate (JC-1 red), and green monomer (JC-1 green) at 485 nm/595 nm or 485 nm/525 nm (excitation/emission), respectively. The ratio of JC-1 red (595 nm)/JC-1 green (525 nm) was calculated as relative mitochondria membrane potential (ΔΨ_m_).

#### Measuring cellular oxygen consumption

Exponentially growing cells (1.5 x 10^6^) were suspended in the respiration buffer containing 20 mM pH 7.1 HEPES (Alfa, Cat. A1477718), 250 mM sucrose, 10 mM MgCl_2_, 2 mM phosphate, then added to the oximetry chamber. Permeabilization of the plasma membrane was carried out by adding Digitonin (25 μg/ml, Biosynth. Cat. D3200) to the chamber under constant stirring. Oxygen consumption was monitored at 30°C with 5 mM succinate (Alfa Aesar, Cat. AA33386-30) as the complex II substrate using the Clarke-type oxygen electrode (Rank Brothers Ltd) following the manufacturer’s instructions.

#### Measuring cellular ATP generation

Cellular ATP content was determined using a luciferase-based ATP assay kit (Molecular Probes, Cat. A22066). Cells were rinsed twice with cold PBS and 40 μL of cold 0.5% trichloroacetic acid (TCA, VWR. Cat. VW3928-2) was added and incubated on ice with shaking for 20 min. Cells were supplemented with 140 mL of 250 mM Tris-Acetate (pH 7.75) per sample, and 10 μL of this cell suspension was mixed with 90 μL of ATP Assay Solution (20 × reaction buffer, 0.1 M DTT, 10 mM Luciferin, 0.25 μL luciferase; Invitrogen). ATP levels were determined using a Turner TD20/20 Luminometer (Promega).

#### LDR of primary breast epithelial cells

Breast tissue biopsies from three healthy individuals with reduction mammoplasty surgeries were collected under standard procedure with consent and the mammary epithelial cells were isolated and cultured following the established protocol.^66^ The primary cultured epithelial cells were pooled and passaged for 2–3 times and treated with sham or LDR (10 cGy) and cell lysate was harvested 12 h after treatment for analysis of induced Per2 expression by western blot.

#### Measuring clonogenic survival

Cells constantly passaged every two days to 80% confluency were exposed to LDR (10 cGy), LDR or LDR 12 h before HDR (5 Gy) and 800 cells were seeded into each well of 6-well plates. Both irradiated and control cells were cultured for 10 to 14 days and colonies were stained with Coomassie blue. The colony with more than 50 cells were counted. The colony images were obtained by Nikon microscope. The clonogenicity were calculated by the percentages of colonies formed from seeded cells in each group.

#### siRNA-mediated Per2 inhibition

siRNA against human PER2 gene was designed and synthesized with the Silencer siRNA Construction Kit (Ambion, Cat. 1620). The primers used to synthesize the targeted and scrambled siRNAs for PER2 were as follows: Scramble: 5-AAATATGTGCGTACCTAGCTTCCTGTCTC-3′, and targeted Per2: 5′-AATGAAGAGTATTACCAGCTGCCTGTCTC-3′. Cells were seeded to achieve 30–50% confluency on the day of transfection and siRNA transfection was conducted using Lipofectamine RNAiMAX reagent (Invitrogen, Cat. 13778-100). Briefly, cells were consistently passaged 2 times before the transfection and seeded into 60-mm cell culture dish one day before the transfection with a 30–50% confluency. After overnight transfection, half amount of fresh medium was added directly into the transfection culture medium and the culture was kept for another 24 h. Scrambled RNA Duplex (Ambion, Austin, TX) served as the specificity control.

#### Measuring cell proliferation

MTT (Sigma, Cat. M−2128) was applied to monitor cell proliferation treated with LDR, HDR or LDR 12 h before HDR in WT MCF-10A cell and siPer2 transfected MCF-10A cells. Briefly, 0.8×10^4^ cells/well were seeded in 96-well plates and incubated for 48 h. Then, the medium was replaced with 100 μL of fresh medium containing 0.5 mg/mL MTT reagent for further 4 h incubation. The medium was removed, and the formazan crystals were solubilized by adding 100 μL of Dimethyl Sulfoxide (DMSO, Sigma, Cat. D2650) for 30 min. The absorbance of the dissolved formazan crystals was recorded using the microplate spectrophotometer (Molecular Devices) at 540–570 nm.

#### Co-immunoprecipitation

Proper antibody was incubated with Protein A or G magnetic beads for 1 h at RT and the protein extracts (500 μg) in 500 μL of 1x RIPA buffer were incubated with antibody coupled magnetic beads on rotator overnight at 4°C. Then, the captured proteins were eluted and denatured by loading buffer with boiling at 95°C for 10 min. The supernatant was applied to SDS-PAGE that was followed by western blotting. The antibodies applied in co-IP were anti-Per2 antibody (Cat. 611138), anti-pGSK3β (Santa Cruz; Cat. 5558T), V5-tag antibody (Proteintech; Cat. 66007-1), p-serine (Cat. 05-1000X) and p-threonine (Cat. SAB5600203).

#### Transfection of Per2-expressing plasmids

Human Per2 cDNA vector pLenti6.3/V5-DEST was kindly provided by Dr. Min Gyu Lee from The University of Texas MD Anderson Cancer Center. Lentiviral particles were packaged in HEK293T cells according to the protocol from Addgene. The stable Per2 overexpression 293T (Per2-OE) was constructed by infecting 293T cell with packaged lentiviruses according to published protocol. GSK3β^wt^ and GSK3β^S9A-mut^ plasmids were derived from Addgene and transfected with the Per2-OE 293T cells following the protocol of turbo reagent transfection (Thermo Scientific, Cat. R0531). The transfected cells were cultured for 24 h before LDR (10 cGy) and cells were collected 12 h after LDR for immunoprecipitation.

#### Analysis of Per2-promoter transactivation

To measure the basal and LDR-induced transcription factors, we utilized plasmid containing the Per2 promoter region (pGL3-basic-mPer2) and included a β-catenin predicted binding site. Cells were co-transfected with pGL2-basic-Per2-2 and Renilla Luciferase-Pol.III by turbo reagent for 24 h then treated cells with LDR. Luciferase activity was measured 12 h after LDR by using 20 μL of total cell lysates and 100 μL of luciferase assay reagent (Promega, Cat. E1483) as described previously [12, 36]. Equal amount of cell lysates was used for the assessment of Renilla activity to normalize luciferase activity.

#### Protein degradation analysis

Chase assay was performed to determine the stability of pGSK3β and active β-catenin in Per2^wt^ and Per2^def^ mouse BMMNCs treated with LDR. Briefly, 5×10^6^ cells were seeded in 100 mm plate and incubated for 24 h before receiving LDR treatment. After 12 h, the medium was replaced with 3 mL of fresh medium containing 30 μg/mL cycloheximide for further incubation. The cells were collected at different time points (0 h, 1 h, 2 h, 4 h) and total cell protein was quantified by BCA Protein Assay kit and western blots performed.

#### Nuclear and cytosolic Per2 and β-catenin level

Western blot was used to investigate Per2 and β-catenin in nuclear and in cytosolic fractions. Cells were digested using lysis buffer (10 mM HEPES, 10 mM KCl, 1.5 mM MgCl_2_, 0.5 mM DTT, 1% IGEPAL 630) with protease inhibitor cocktail (Sigma, Cat. P8340). Cells were incubated for 7 min on shaker at 4°C and transferred to 1.5 mL centrifuge tube, followed by centrifuging at 12,000 rpm for 1 min. The supernatant obtained correspond to the cytosolic fraction and the pellet was washed by washing buffer (10 mM HEPES, 10 mM KCl, 1.5 mM MgCl_2_, 0.5 mM DTT). The pellet was then resuspended in extraction buffer (10 mM HEPES, 410 mM KCl, 1.5 mM MgCl_2_, 0.5 mM DTT, 0.2 mM EDTA, and 25% Glycerol). The supernatant was saved as nuclear fraction after 14,000 rpm centrifugation at 4°C for 10 min. Total protein content was measured by BCA Protein Assay kit and western blot was performed.

### Quantification and statistical analysis

#### Statistical analyses

All statistical analyses were using GraphPad Prism 8.0. Software (GraphPad Software, Inc., San Diego, CA). Data were presented as mean ± SEM For comparison of survival curves, a log rank test was applied. Other results were analyzed by the two-tailed student t-test and one way ANOVA with data considered significant at p <0.05. All data were presented as the standard error of the mean with at least three individual experiments.

## Data Availability

• The RNA-Seq data of Per2Def versus Per2wt mouse cells have been deposited at the NCBI Gene Expression Omnibus (GEO) and are available at JJ Li’s lab. The accession number and access information: Gene Expression Omnibus accession number: GEO accession GSE128418: •
https://www.ncbi.nlm.nih.gov/geo/query/acc.cgi?acc=GSE128418
• For Reviewer Access, the following secure token has been created to allow review of record GSE128418 while it remains in private status: chyrgocqjnibpwd. The DOI is also listed in the key resources table.

## Supplemental information

Document S1. Figures S1–S10

Document S2. KO_vs_WT_HSC_Cuffdiff_Isoforms

Document S3. KO_vs_WT_HSC_Cuffdiff_Genes (1)

Document S4. KO_HSC_RNA-Seq_Cufflinks

Document S5. WT_HSC_RNA-Seq_Cufflinks
