# Supplementary material for: PERIOD 2 regulates low-dose radioprotection via PER2/pGSK3β/β-catenin/Per2 loop
Source: iScience. 2022 Nov 9;25(12):105546. doi: 10.1016/j.isci.2022.105546 (PMC9708791; doi:10.1016/j.isci.2022.105546)
Supplement: Document S1. Figures S1–S10 [file mmc1.pdf]

iScience, Volume *25*

Supplemental information

PERIOD 2 regulates low-dose radioprotection via PER2/pGSK3β/β-catenin/Per2 loop

Aris T. Alexandrou, Yixin Duan, Shanxiu Xu, Clifford Tepper, Ming Fan, Jason Tang, Jonathan Berg, Wassim Basheer, Tyler Valicenti, Paul F. Wilson, Matthew A. Coleman, Andrew T. Vaughan, Loning Fu, David J. Grdina, Jefferey Murley, Aijun Wang, Gayle Woloschak, and Jian Jian Li

Supplemental Data

PERIOD 2 Regulates Low Dose Radioprotection via PER2/pGSK3β/β-Catenin/Per2 Loop

Aris T. Alexandrou, Yixin Duan, Shanxiu Xu, Clifford Tepper, Ming Fan, Jason Tang, Jonathan Berg, Wassim Basheer, Tyler Valicenti, Paul F. Wilson, Matthew A. Coleman, Andrew T. Vaughan, Loning Fu, David J. Grdina, Jefferey Murley, Aijun Wang, Gayle Woloschak, and Jian Jian Li

Supplementary Figures: Figure S1 to S10

Other supplementary materials for this manuscript include the following: none

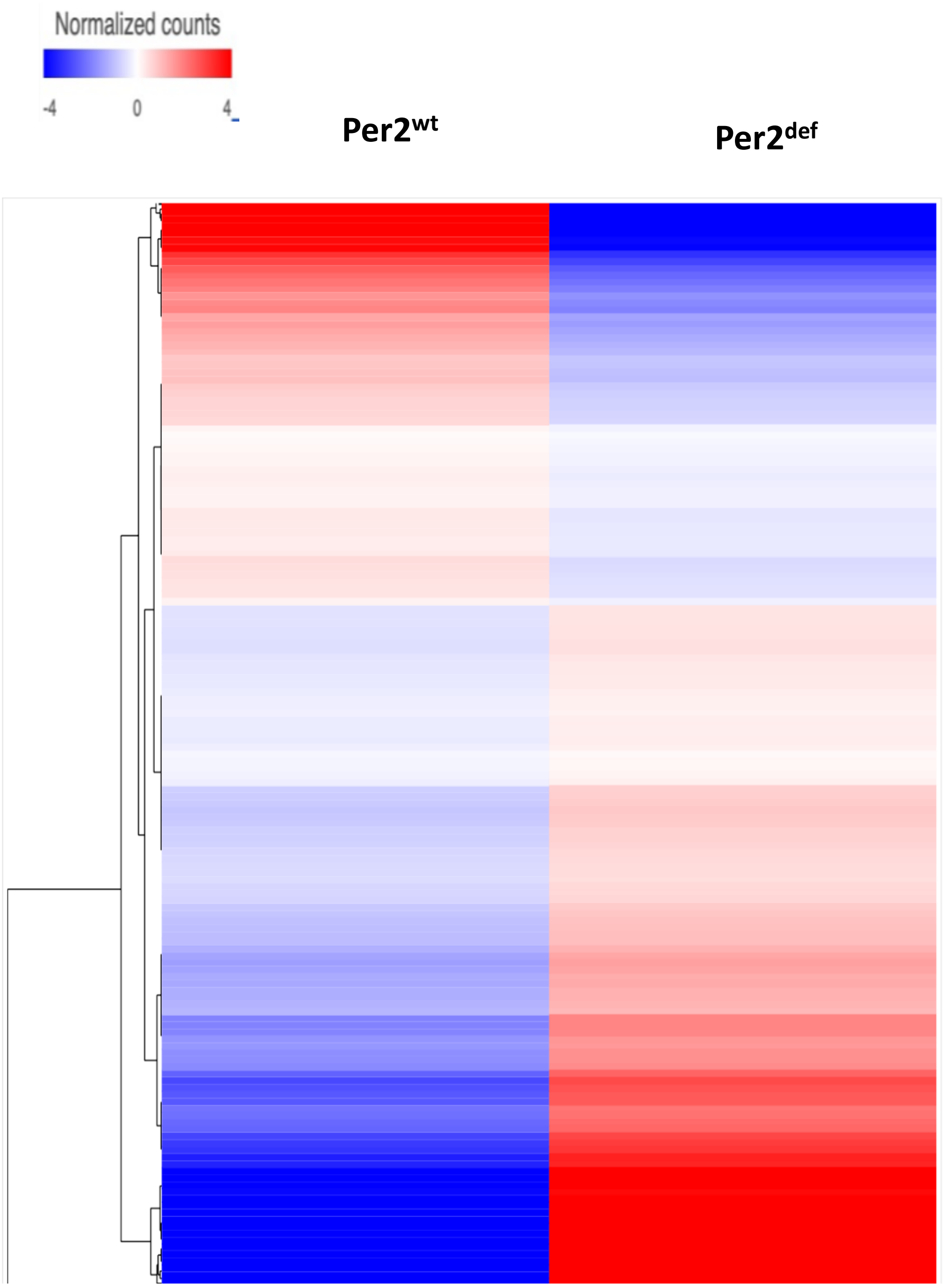

Figure S1. RNAseq profiling of Per2^{wt} and Per2^{def} bone marrow cells, Related to Figure 2. Differentially-expressed genes (1.2X fold change) in BMpHSCs (BM-LSK-pHSCs) isolated from bone marrow cells of Per2^{wt} and Per2^{def} C57BL/6 mice.

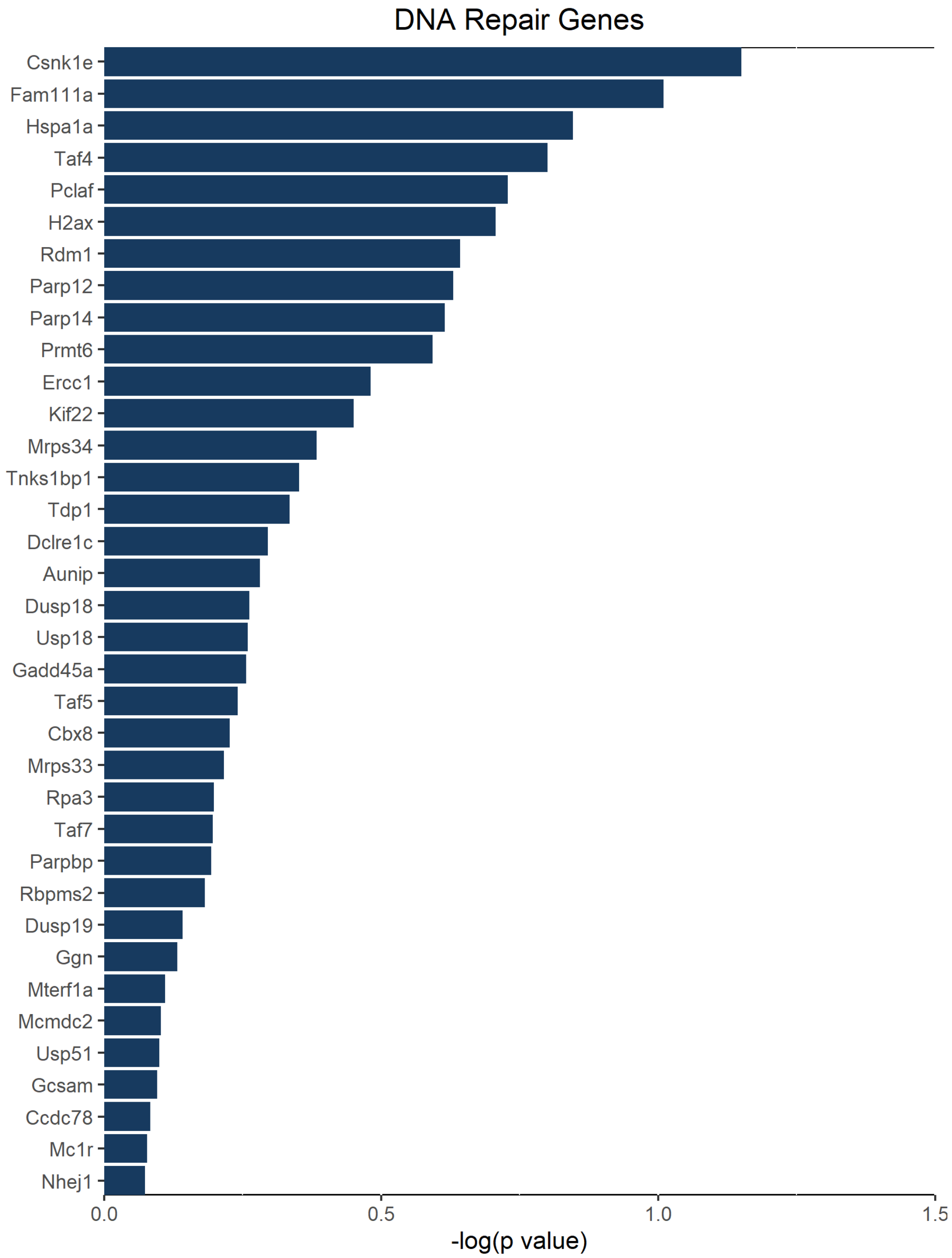

Figure S2. Per2-associated DNA repair genes in Per2^{wt} BMHSCs versus Per2^{def} BMHSCs, Related to Figure 2. Expression levels of PER2 related DNA repair genes that are silenced in Per2^{def} BMpHSCs.

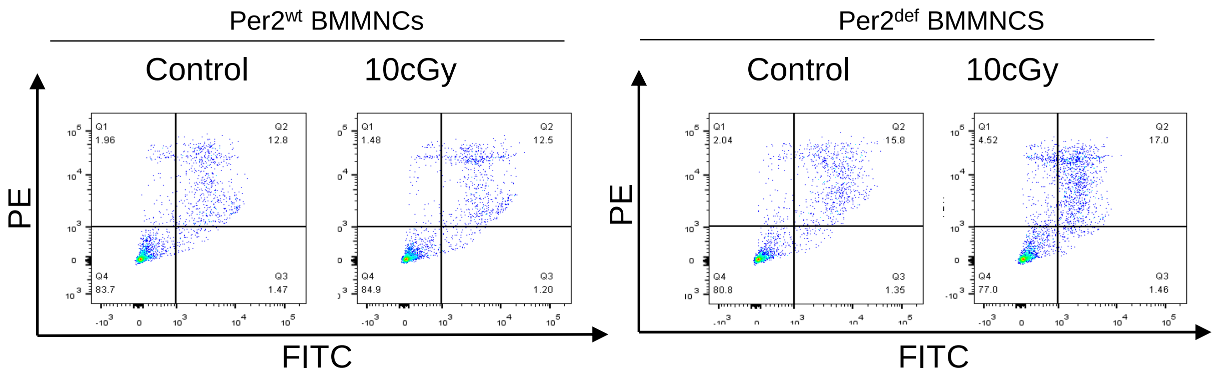

Figure S3. LDR induced adaptive response in Per2^{wt} and Per2^{def} BMMNCs, Related to Figure 2. Representative flow cytometry analysis of LDR induced apoptosis in BMMNCs isolated from Per2^{wt} and Per2^{def} mice 24 h after LDR (10 cGy).

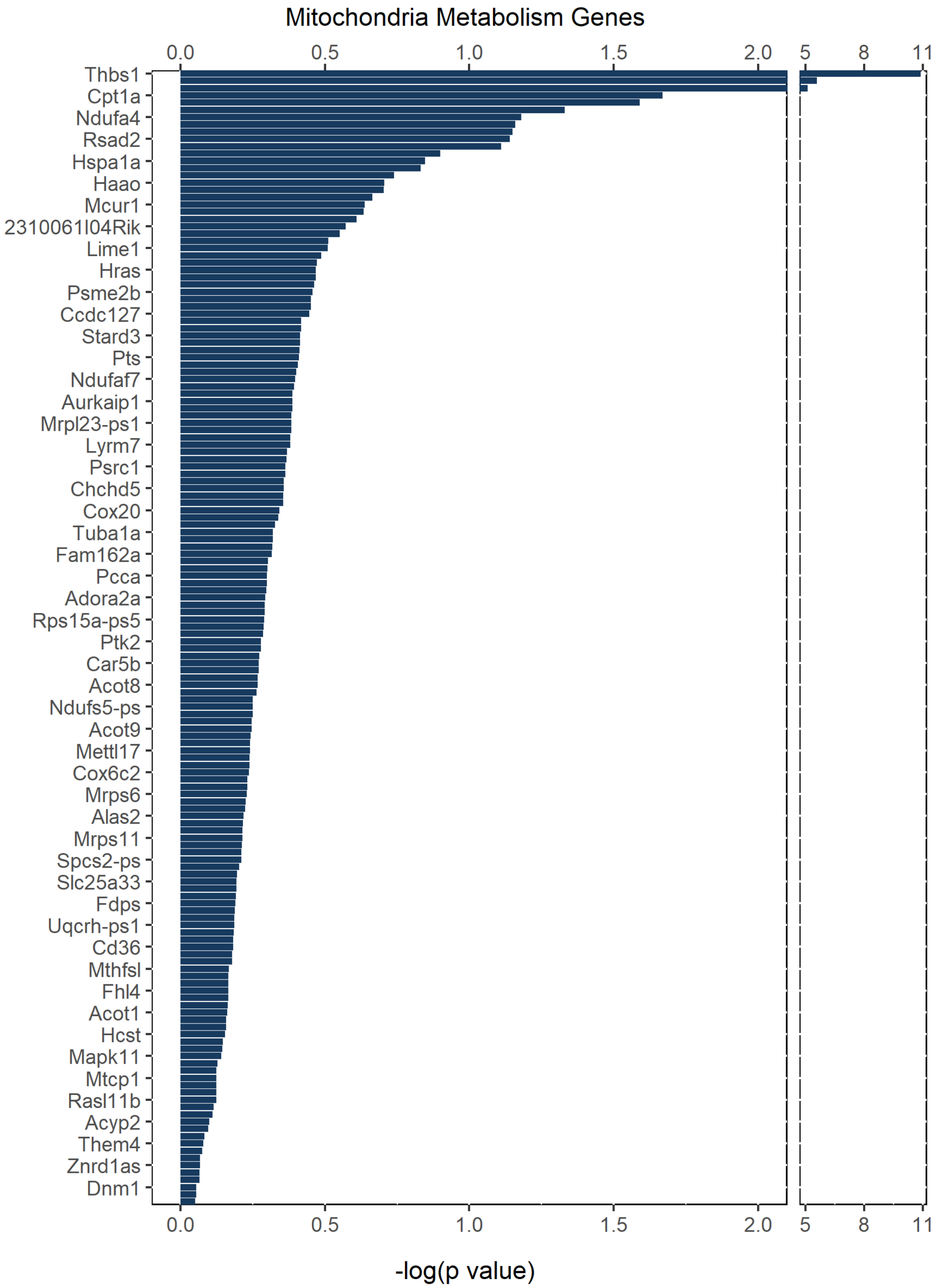

Figure S4. PER2 related effector genes involved in mitochondrial metabolism in in Per2^{wt} BMpHSCs versus Per2^{def} BMpHSCs, Related to Figure 3. A cluster of PER2 related genes involved in mitochondrial metabolism genes silenced in Per2^{def} BMpHSCs.

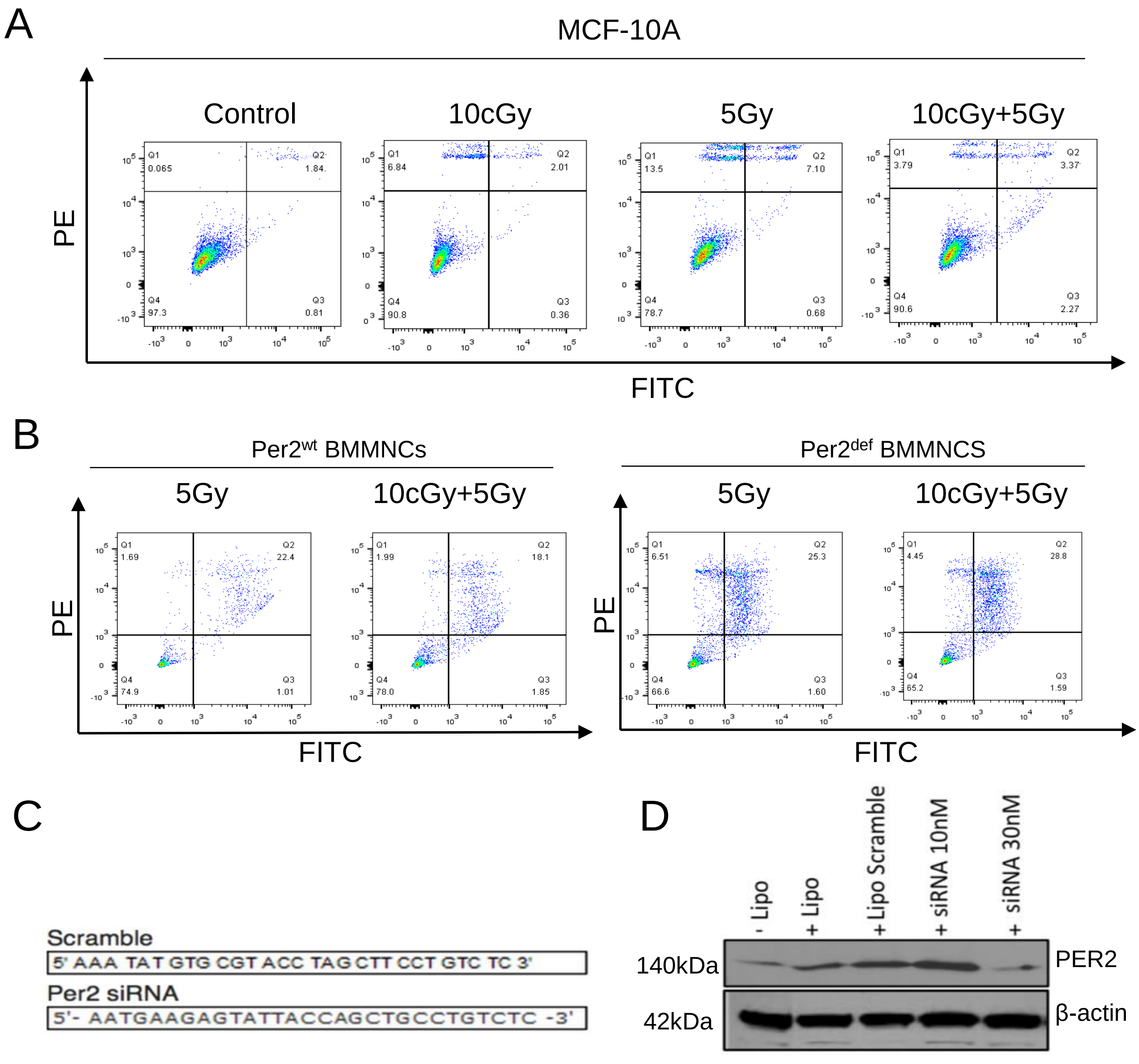

Figure S5. PER2 mediated radioprotection in LDR treated cells, Related to Figure 4. (**A**) Apoptosis of MCF-10A cell exposed to LDR (10 cGy), HDR (5 Gy) or LDR + HDR doses of radiation. (**B**) Apoptosis of $Per2^{wt}$ and $Per2^{def}$ BMMNCs exposed to HDR (5 Gy) or LDR + HDR doses of radiation. (**C**) Human siRNA sequences for scramble and targeted Per2. (**D**) Immunoblot of Per2 in LDR (10 cGy) treated MCF-10A cells 24 h after transfection with scramble Per2 siRNA or two concentrations (10 nM and 30 nM) of human Per2 siRNA.

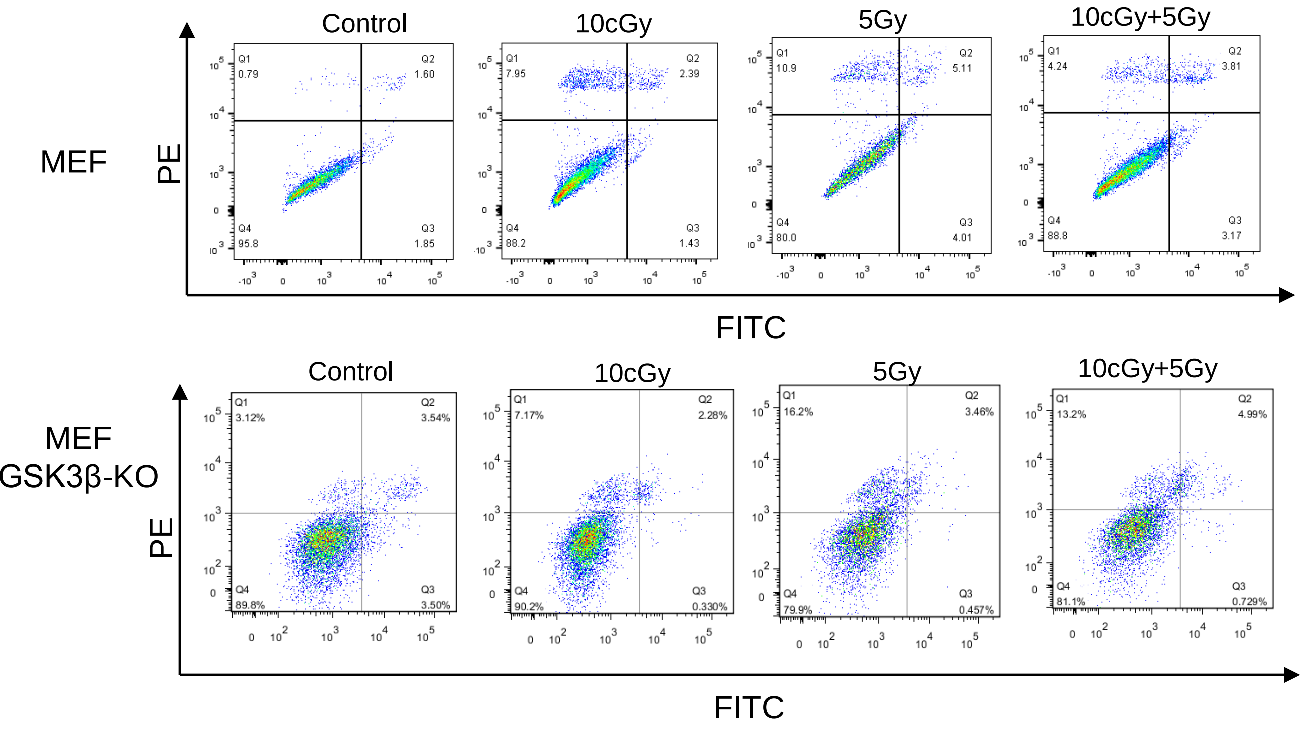

Figure S6. GSK3ß participates in LDR induced radioprotection, Related to Figure 5. Apoptosis of GSK3ß^{wt} versus GSK3ß^{ko} mouse embryonic fibroblasts (MEF) treated with LDR (10 cGy), HDR (5 Gy) or LDR 16 h later + HDR.

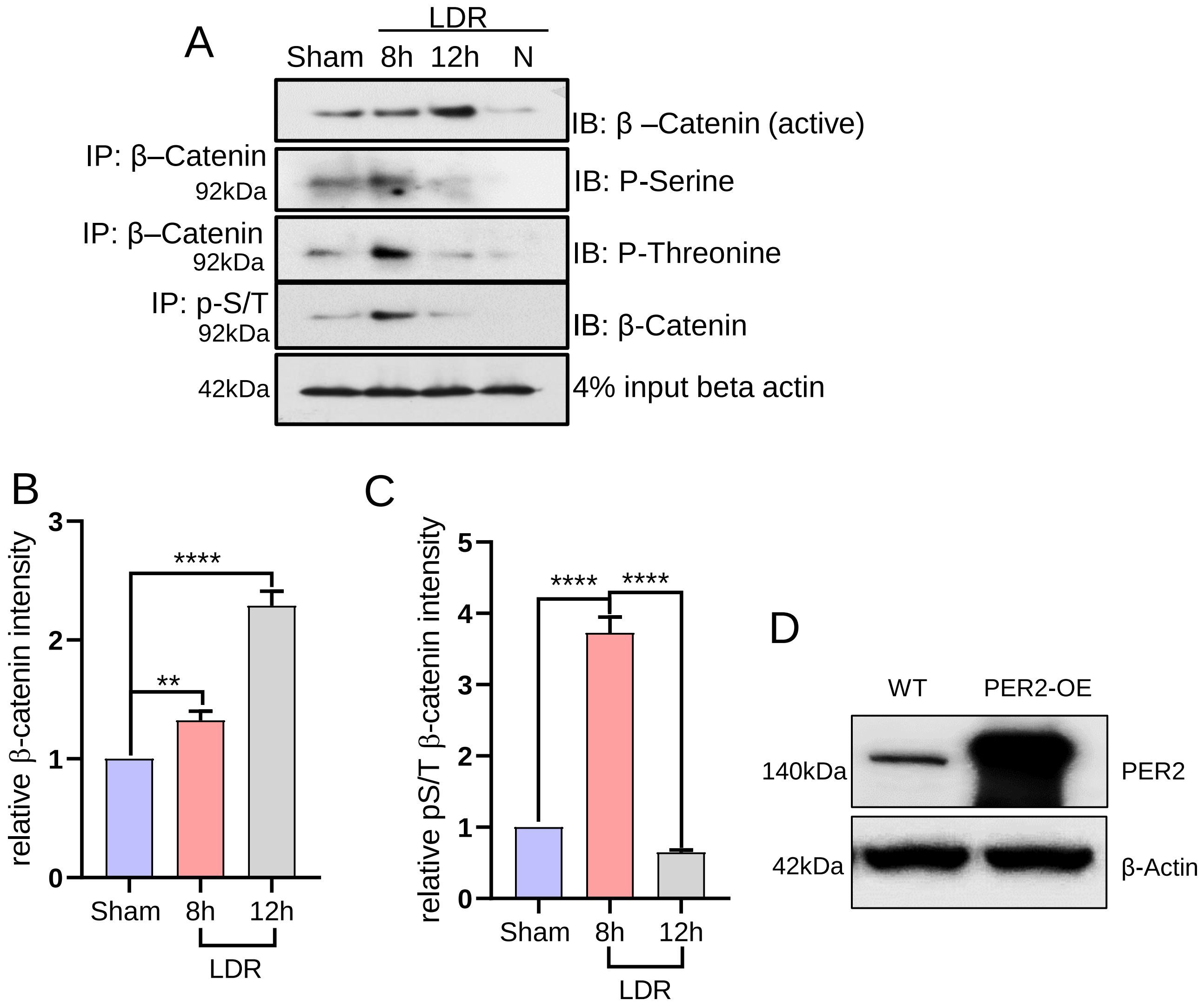

Figure S7. LDR enhanced active β-catenin expression, Related to Figure 6. (**A**) Active ß-catenin peaked 12 h in LDR treated MCF-10A cells detected by western blot of ß-catenin. Phosphorated ß-catenin was detected by immunoprecipitation (IP) of ß-catenin followed by immunoblotting (IB) with anti-p-Serine/Threonine (anti-pS/T) or IP with anti-pS/T followed by IB with anti-ß-catenin (N= negative control without antibody). **(B,C)** Relative active (B) and inactive (C) ß-catenin in LDR treated MCF-10A cells 8 h or 12 h after LDR quantified with Image J and normalized with β-actin levels. Data are represented as mean ± SEM, n = 3, **P < 0.01, ****P < 0.0001, ANOVA two-way test was applied. (D) Identification of overexpressed Per2 in 293T cells with β-actin as loading control.

A

Table A. TCF/LEF binding sequences in mouse per2 promoter region

LEF-1 / TCF-1A	T02905/T00999	-597-589	CTTTGGCC
		-703-690	CTTTCCTTTGTAT
		-1000-991	CCTTTGGAC
		-1397-1389	CTTTGGAA
		-1544-1536	CTTTGGGT
		-1802-1794	TTCCAAAG
		-2397-2371	TCCCAAAG
TCF-4	T02918	-2018-2006	GCCTTTAATCCC
TCF-1(P)	T01109	-114-103	TAAAGAGAGCG
		-466-456	AATGAGAGGG
		-513-503	ATCCAGAGGG
		-743-732	TTCTCTCCCT
		-1456-1445	AACCAGAGGTG
		-1608-1598	TACTCTTGTT
		-1820-1810	AACCAGAGAC
		-1923-1913	ATACAGAGAA
		-1996-1986	AGGCAGAGGC
		-2246-2236	ATCTCTTCTT
TCF-2	T01110	-2016-2006	CTTTAATCCC
		-2273-2263	CTTATAACCC
TCF-3	T02857	-1011-1000	CCTCCTTTGAC
		-2627-2616	ATAAAAGTAAG
		-2930-2919	TATTCTTTAAT

Table B. TCF/LEF motif distribution in mouse per2 promotor

B

-3000
CCTAAAACCTCCTCCTAGGACCCACTCCTCCAAGGCCTCACCACTTCCCAGTGATAGCATGTTGGTAACCTATTCTTTAATGTAC
AGGTGTTTGAAGACATTCCAGAGCCAAATTATAGCAGGATTTCACCTTGAGTATGGGGATCACCTGGAGTGTGGAGACTACT
CCAGGTCCAAGGATCAATCATCTCAGACGTGGGACAGTTTATAGTTTGAACGTGATATATTTCCCACCAGCTCTGGTGTTTGAG
CACTTAGCCCTTCGGTGTTGGTGTCATTTTTGGAGGTTGTAGAACCTCTGAGGGAACCACATACATGGTGTACAGATATACATG
CAGTCAAAATATACATACACACAAAAATTTTAAGAAAATAAAAGTAAGTGATACAGGGCATTGCCTCAGGCATTGGGTGTTAT
ATCAGACATGAGGGACACTCAAGACATGGGGAGCACCTCGTAGGTAGTGCATCACCTTGGACACAGTTGTCTTGTGACAAAAT
GTCTTCCTGTGGGCCCCTTCAAAACTGTGAGAGATATGAGTGGCCACAAAACCCTGGAAAGAATCTCAAACCCTGGAAAGAGT
CTCAGAAAACCTTGATCCACAACTCAATTCCCAAGTTCCCAAAGCTTGATCTACAGCACCAACTCCCAGAACTGTCATCCACATG
ACTACCCATCCCCCACCAAAAGACACAACTCTTATCTCAGTCAAGCCATTGGCTGTCCTTATAACCCTGAAACTTGATGCTTCAA
TCTCTTCTTGCATAGCATGGCAGCAAATAGTTTTAGTCTGGGAGGAGGCTTTTTTAAAAACTGTGTCTTCAGGGCTGAAGAGAT
AGTTTAGCAGTTAAAAATACATATTGCCTTTACGGAGGACTCAGGTTTTGGTTTCCAGCACCCATGTGGCAGCTCACAACAATC
TATAACTCAAGCCAGTGTGGGATACAGTGTAAAAATCCAAACCGGGTGTGGTGGCGCACGCCTTTAATCCCAGCACTCGGGAG
GCAGAGGCAGGCGGATTTCTGAGTTCGAGGCCAGCCTGGTCTACAAACTGAGTTCCAGGACAGCCAGGACTATACAGAGAAA
CCCTGTCTCAAAAAAAAAAAAAAAAAAAAAAAAAAAAAAAAAAAAAAAAAGAAAGAAAGAAAGAAAGAAAAGAAAGAA
GAATCCAACCAAAACCAGAGACAAATTAGATTCCAAAGAGTTCTAGAGTCCCCACAGTATTGTGTTTCCAAAAGAATTGATTAC
TATGTCTTTCTAGACTTACTGGTGGAAATAGAAAAGGCTAAACTTAGGGAGGAACTGTGACATTACATTGCAGGCCAAATGTA
GAGCAAGACAAGAAAAAGCTGAGCATGAAGGAGACTCTGCCAGGTGGATGAGCTGTGTACTCTTGTTTCCAGAACAATGTAG
CCACCATTGCCGTCAATGTAAGCGAGGAAACAAAAGGCCCTTTGGGTGTGTGCAGGGTGCAGCTTGGCCCAGCTCTGCTCAGT
GTTTGTGTGTGTTGGGGAGTGTGGTGAGGTGTCAGTGTCAGAGGAACCAGAGGTGCTGCCCTGCCCCCTGCAGTGTGAGTCA
ACATCTGGCTTCCCAGGGCTTCTTTGGAAAGGGCTGCTGAAATGAACTTAGTCTCTGCCCCCATCTGCATCTGAGGAATTGCAT
GCCTGTCCTGCCAGGCAGACAGAAAGAAGTAGCTCCCACACGGAATTCTTGAATGTGGGTTAGCCGGCTGTGTACACCAGCA
GCTCAGTTTGTTAGCAGACTTCTGTTGCTAATGTTTGCCTCCTTTCCATTCCTGGTTCCTAGGACACCCCAGGGGAAGATTCAGA
GTAGTGGATGCTACTAGGCTTCAAGTTCCCTGGCAATGACAAATGACCTTTTTACCCTTGGAAGACGTGACAAGCTTGCCTTCT
CCATCACACCTTGCATGAGTCTTTAGGTTGTTCTCTGTCAGCCTCAAACCCGCTCCGAGGAAACTTCTACTCCCTCCTTTGACCCT
TTGGACAGGAGCCTGAACGCTTTAGTAGGCTTCCAGACAGTGCTCTTGAAAGAACCAAATAGCTTCAACCAAGGTTCCACAGG
GGCAGGGCTGTCCTATCACTGGAGGAGTACCCTCCCCTGACTAGCTAGTGTCTGTAGCTTCCACTTCAGAAAAGCCCTGCTGTT
CCAGATGCACACCCCGCTTCCATAGTTCCTGTAAGGTTAATAAACTACACCACCGCATTTGGTTAAGCTTCCCTGTAGAACGTCA
GTCTTCTCTCCCTATGTGATTGAGGGCAGGAAGAAATCACTTCTTTCCTTTGTATCTCTGCACGGCAATTATGACCTTATTTCCTG
AATCAACACTAACTAGCAACACGCAGTTTCAGAAACAAGAAAGGCTAAGTGGGAGTTTTGTGCTTTGGCCCATCTGGAATGAC
GGTCAGCCTGGGGGGCCTGTCCTAGGGTCACCCAGCCTGTCCTGGGAAGGTGCTCAGCAGCAGATCCAGAGGGGCCGTCCTA
TTTGCCCTCAAGCGTCTCGCCATGAATGAATGAGAGGGGAAATGAATGAACTGGGCTGGATGAGCGAAAGGTGTCAGCAGA
GAGCATTCTCGGTCCTTCGGATTACCGAGGCTGGTCACGTCGTCGCAGGTGATAGGCCGGTGGCCCTGGTCTCTGCCGGCTGT
GAGTTGCGCAGCGGCCAAGCACCATTCCCCCGCGCCGCAGTGGTACGCGCCACTCCGGGGCTGCACGAGCGGGCCACCGCCG
TGCCAGGTGAATGGAAGTCCCGCAGGCCGGAAGTGGACGAGCCTACTCGCCCGGGCGCGGGGGGCGCAAGAGCGCGCAG
CATCTTCATTGAGGAACCCGGGCGGCGAACATGGAGTTCCATGTGCGTCTTATGTAAAGAGAGCGACGGGCGTCTCCACCAAT
TGATGAGCGTAGCTCTCAGGTTCCGCCCCGCCAGTATGCAAATGAGGTGGCACTCCGACCAATGGCGCGCGCAGGGGCGGGC
TCA -1

LEF-1 [T02905]/ TCF-1A [T00999] TCF-4 [T02918] TCF-1(P) [T01109] TCF-2 [T01110] TCF-3 [T02857]

Figure S8. The PER2 promoter contains TCF transcriptional binding site, Related to Figure 6. (A) Table A: Predicted TCF/LEF binding site sequence. (B) Table B: Schematic presentation of mouse Per2 promoter region enriched with TCF/LEF binding motifs.

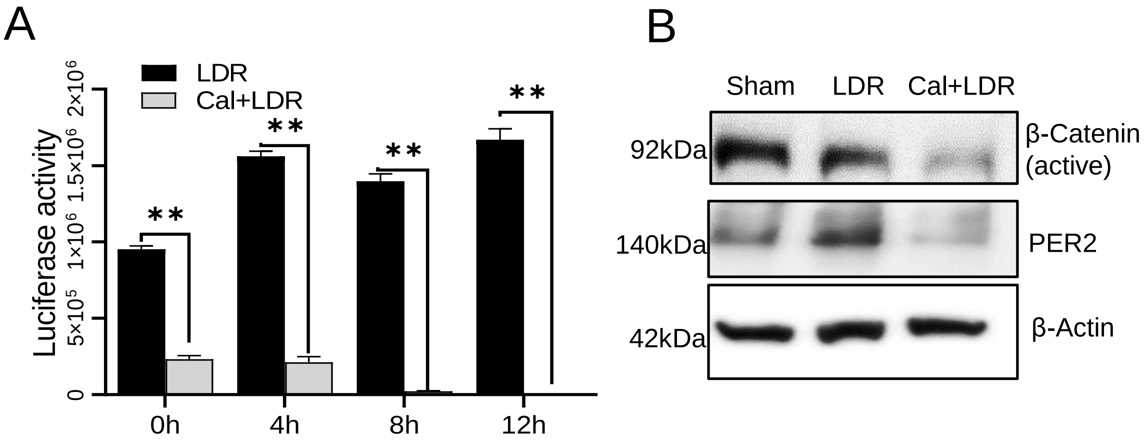

Figure S9. PER2 regulates GSK3ß/ß-catenin pathway in LDR induced radioprotection function, Related to Figure 6. (**A**) Per2-Luciferase reporter activity measured in MCF-10A cells treated with LDR or LDR + TCF/LEF inhibitor 0.1 µM Cal (Cal: homologous to transcription binding site on the promoter of β-catenin). Luciferase activity was measured 0, 4, 8 ,12 h after Cal treatments. Data are represented as mean ± SEM, n = 3, **P < 0.01, Student's t test. (**B**) Per2 expression was inhibited with ß-catenin inhibitor measured by western blot in MCF 10A cells 12 h after LDR or Cal treated 1h followed by LDR or sham irradiation. ß-actin was included as a loading control.

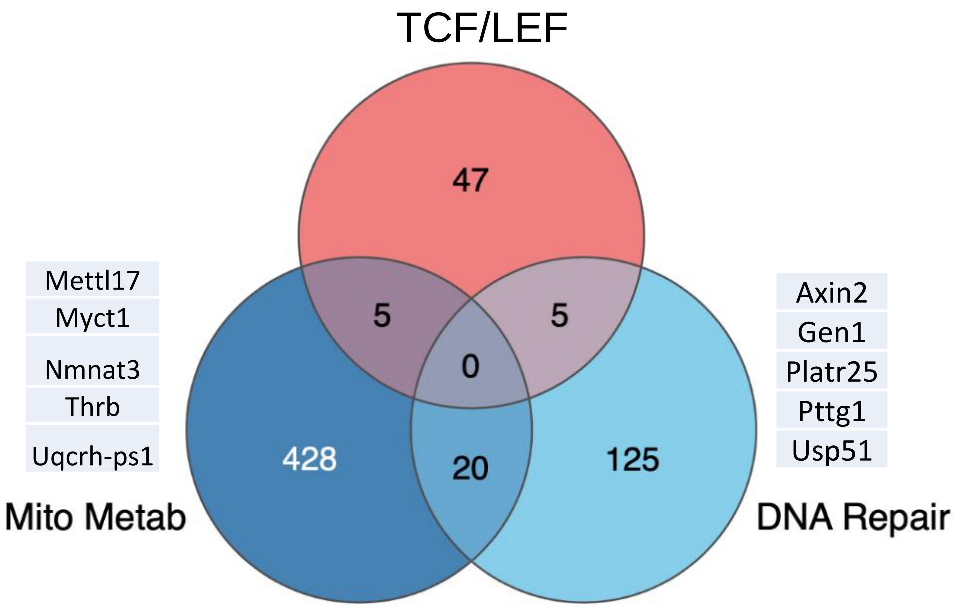

Figure S10. Venn Diagram showing intersection of TCF regulated genes, Related to Figure 10. TCF responsive genes for DNA damage repair and mitochondrial metabolism were identified with 1.2-fold cutoff differentially expressed in BMpHSCs of Per2^{wt} and Per2^{def} C57BL/6 mice.
